# O-GlcNAcylation determines the biological function of YTHDF proteins

**DOI:** 10.1038/s41556-023-01258-x

**Published:** 2023-11-09

**Authors:** Yulin Chen, Ruixi Wan, Zhongyu Zou, Lihui Lao, Guojian Shao, Yingying Zheng, Ling Tang, Ying Yuan, Yun Ge, Chuan He, Shixian Lin

**Affiliations:** 1 Zhejiang Provincial Key Laboratory for Cancer Molecular Cell Biology, Life Sciences Institute, Zhejiang University, Hangzhou, China.; 2 Shaoxing Institute, Zhejiang University, Shaoxing, China.; 3 Department of Chemistry, Department of Biochemistry and Molecular Biology, and Institute for Biophysical Dynamics, The University of Chicago, Chicago, IL, USA.; 4 Howard Hughes Medical Institute, The University of Chicago, Chicago, IL, USA.; 5 Institute of Chemical Biology, Shenzhen Bay Laboratory, Shenzhen, China.; 6 Department of Medical Oncology, The Second Affiliated Hospital, Zhejiang University School of Medicine, Hangzhou, China.; 7 Cancer Center, Zhejiang University, Hangzhou, China.

## Abstract

N^6^-methyladenosine (m^6^A) is the most abundant internal mRNA nucleotide modification in mammals, regulating critical aspects of cell physiology and differentiation. The YTHDF proteins are the primary readers of m^6^A modifications and exert physiological functions of m^6^A in the cytosol. Elucidating the regulatory mechanisms of YTHDF proteins is critical to understanding m^6^A biology. Here, we report a mechanism that protein post-translational modifications control the biological functions of the YTHDF proteins. We find that YTHDF1 and YTHDF3, but not YTHDF2, carry high levels of nutrient-sensing O-GlcNAc modifications. O-GlcNAc modification attenuates the translation promoting function of YTHDF1 and YTHDF3 by blocking their interactions with proteins associated with mRNA translation. We further demonstrate that O-GlcNAc modifications on YTHDF1 and YTHDF3 regulate the assembly, stability, and disassembly of stress granule to enable better recovery from stress. Therefore, our results discover an important regulatory pathway of YTHDF functions, adding an additional layer of complexity to the post-transcriptional regulation function of mRNA m^6^A.

## Introduction

The most prevalent internal mRNA modification in mammals, m^6^A, occurs at tens of thousands of sites throughout the transcriptome at a frequency of 0.15–0.6% of all adenosines. Up to 60% of transcripts contain at least one m^6^A modification site ^1–5^. m^6^A is installed by the METTL3/METTL14 methyltransferase complex ^6–11^ and is removed by the demethylation enzymes ALKBH5 and FTO ^12, 13^. Given its high abundance in transcripts, it is not surprising that m^6^A modification regulates numerous biological processes under physiological and disease conditions ^14–21^. The cellular effects of m^6^A encompass mRNA processing, nuclear export, stability, and translation ^22–28^. These effects can be mediated by m^6^A-binding proteins (reader proteins), which typically contain an m^6^A-specific binding domain and an effector domain that exerts biological functions ^29–31^.

The mammalian YTH *N*^6^-methyladenosine RNA binding proteins, comprising YTHDF1, YTHDF2, and YTHDF3, are the only cytosolic YTH family m^6^A readers and are highly similar in amino acid sequence ^32–35^. Despite extensive studies of YTHDF proteins, there are contradictory reports on the cellular functions of different YTHDF proteins in the literature ^22, 25, 33, 36^. In particular, it is unclear whether YTHDF proteins act in similar or distinct manners, and why YTHDF1/3 proteins may exhibit distinct effects in different cell lines and experimental conditions ^33, 34, 37^. Described in the most prevalent model, YTHDF1 enhances mRNA translation efficiency, whereas YTHDF2 promotes mRNA degradation, and YTHDF3 enhances both translation and degradation of mRNA ^32–34^.

Thus, YTHDF proteins regulate the translation and degradation of m^6^A-modified mRNAs in context-dependent manners ^30^. Recently, a report shows that YTHDF proteins differ in their low-complexity domains, and this provides a molecular mechanism to their distinct functions ^38^. In contrast, some reports argue that YTHDF proteins act redundantly to mediate mRNA degradation but exert no detectable translation-promoting effects ^35, 39^. Therefore, factors that affect functions of YTHDF proteins remain poorly understood.

Here we report that protein post-translational modifications (PTMs) of YTHDF proteins control their biological functions. We find that YTHDF1/3, but not YTHDF2, can be extensively and reversibly modified on the N-terminal low-complexity domains (LCDs) by O-linked N-acetylglucosamine (O-GlcNAc). O-GlcNAcylation is a critical nutrient-sensing PTM that is mediated by a single pair of enzymes, O-GlcNAc transferase (OGT) and O-GlcNAcase (OGA) ^40–48^. The hydrophilic O-GlcNAc modification drastically alters the biochemical properties of hydrophobic LCDs. We show that the O-GlcNAc modification of YTHDF1/3 prevents their interactions with the translational components, thereby inhibiting the effects on promoting mRNA translation. This mechanism is utilized to regulate the translation efficiency of m^6^A-modified mRNAs during cell cycle. In addition, it is tightly regulated in different cell lines and cellular environments. Furthermore, we discover that O-GlcNAc modification increases the dynamics of YTHDF1/3 proteins in stress granules, regulating the assembly and disassembly of YTHDF1/3-containing stress granules. Our findings provide an additional layer of regulation functions of mRNA m^6^A through YTHDF proteins.

## Results

### Distinct patterns of YTHDF protein O-GlcNAcylation

**Figure 1.**
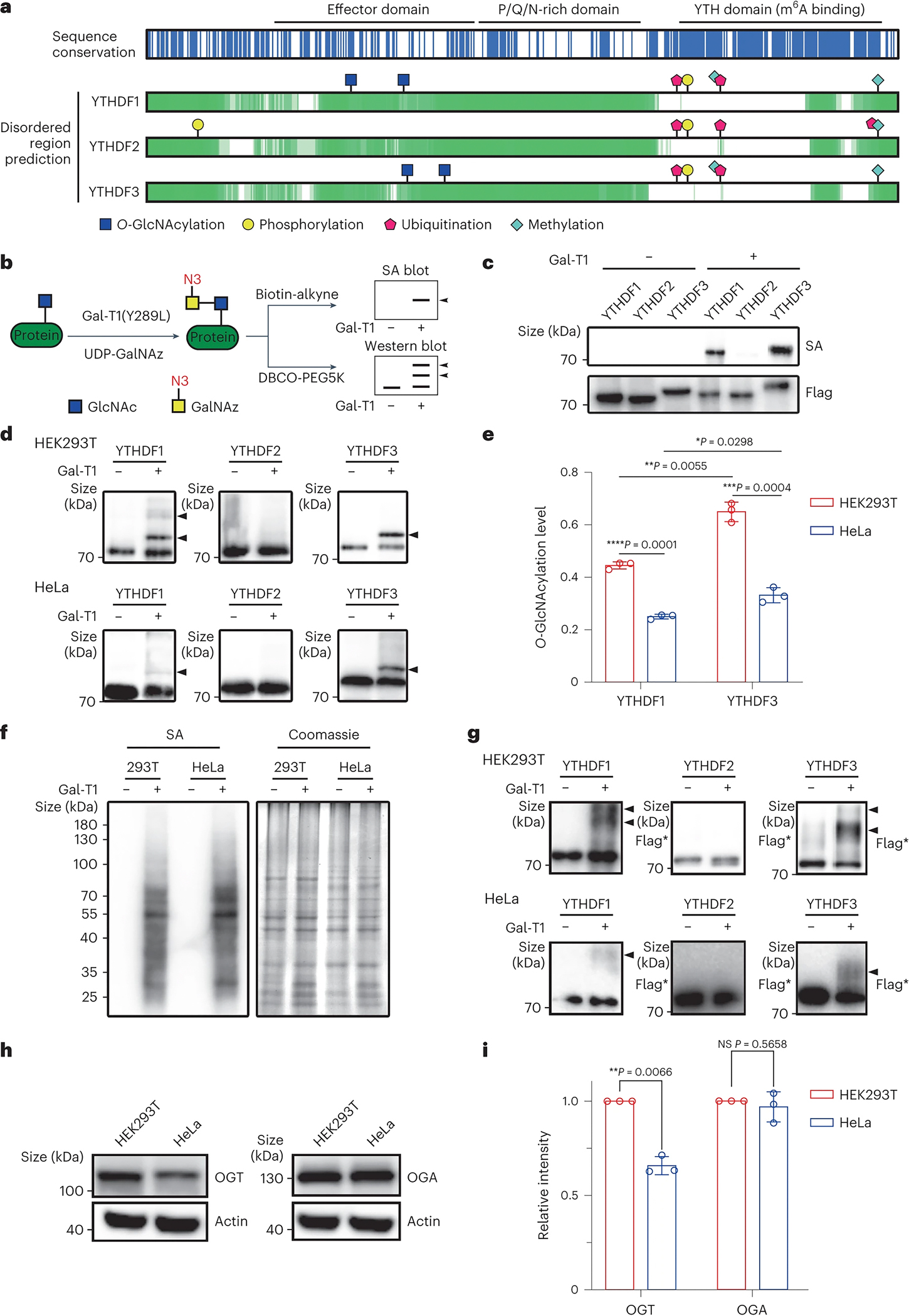
YTHDF1 and YTHDF3 are modified by O-GlcNAc. (a) The three YTHDF paralogs have low sequence conservation (above) and similar intrinsically disorder regions (bottom) along the effector regions instead of YTH domains. By LC-MS/MS identification, YTH domains are modified by similar modifications in all three YTHDFs, but effector domains of YTHDF1 and YTHDF3 are modified by O-GlcNAc. PTM sites identified by LC-MS/MS of YTHDF proteins from HEK293T cells are highlighted with different shapes. Effector domain, P/Q/N-rich domain and YTH domain are annotated. Blue indicates sequence conservation sites. Green indicates predicted disorder regions. (b) Strategy of O-GlcNAcylation analysis using two-step labelling protocol of chemo-enzymatic labelling and CuAAC or SPAAC. First, protein with O-GlcNAcylation is labelled by Gal-T1(Y289L) and conjugated with GalNAz containing azide handler on GlcNAc moiety. Then, the azide group is reacted with biotin-alkyne or DBCO-PEG5K by CuAAC or SPAAC. Finally, biotin signal is detected by streptavidin blot and O-GlcNAcylation stoichiometry is determined with mass-tag by western blot. Besides, shifted band number indicates O-GlcNAcylated site number. (c) O-GlcNAcylation analysis of immuno-precipitated flag tagged YTHDF1, YTHDF2 and YTHDF3 from HEK293T cells. SA denotes streptavidin blot by streptavidin-HRP. (d) O-GlcNAcylation stoichiometry analysis of YTHDF1, YTHDF2 and YTHDF3 in HEK293T and HeLa with indicated antibodies for western blot. The shifted bands pointed by arrowheads are target proteins with O-GlcNAcylation. Two shifted bands indicate O-GlcNAcylation occurs on two sites. (e) Quantification result of O-GlcNAcylation level of YTHDF1 and YTHDF3 in (d). Data are presented as mean ± SD (n = 3 biologically independent replicates). Significance was calculated using two-sided t-test. (f) O-GlcNAcylation analysis of total endogenous O-GlcNAcylation levels in HEK293T and HeLa. Coomassie staining is performed as loading control. (g) O-GlcNAcylation stoichiometry analysis of transfected flag tagged YTHDF1, YTHDF2 and YTHDF3 in HEK293T and HeLa. Flag* denoted samples which were performed O-GlcNAcylation stoichiometry analysis and immunoblotting by anti-flag antibodies. (h) Expression level analysis of OGT and OGA by western blot with actin as the loading control in HEK293T and HeLa cell lysate. (i) Quantification result of expression level of OGT and OGA between HEK293T and HeLa in (d). Data are presented as mean ± SD (n = 3 biologically independent replicates). Significance was calculated using two-sided t-test.

Since protein PTM plays key roles in determining protein functions in a context-specific manner ^49, 50^, we envisioned that PTM patterns might affect YTHDF protein functions, especially in light of their high sequence similarity. To test this hypothesis, we overexpressed and enriched all three YTHDF proteins from HEK293T cells for a thorough identification of protein PTMs using LC-MS/MS (Figure S1a). All common PTMs, including protein phosphorylation, acetylation, methylation, ubiquitination, O-GlcNAcylation, were surveyed and quantitatively analysed (Data S1). As expected, very similar patterns of most PTMs were identified and these modifications were located at highly conserved sequences of all three YTHDF proteins, validating the reliability of the mass spectrometry results (Figure 1a and S1a). Surprisingly, protein O-GlcNAcylation displayed distinct patterns among the three YTHDF proteins (Figure 1a and S1a). O-GlcNAcylations were abundant on YTHDF1/3, whereas no O-GlcNAcylation was detected on YTHDF2. Importantly, O-GlcNAcylation sites were exclusively located at the center of the effector domains in the low-complexity domains (LCDs) of YTHDF1/3, with the peptide sequence surrounding this modification being the least conserved among all three effector domains of YTHDF proteins (Figure 1a and S1a). To validate this discovery, we applied a chemo-enzymatic assay to detect and quantify the levels of O-GlcNAc modification (Figure 1b) ^51–58^. Consistent with the MS results (Figure S1b–c), we detected significant O-GlcNAc modifications on YTHDF1/3 proteins but not on YTHDF2 protein using this chemo-enzymatic assay (Figure 1c).

We next analysed endogenous O-GlcNAc modification in HEK293T cells and found that ~50% of YTHDF1/3 proteins carry O-GlcNAcylation in HEK293T cells (Figure 1d–e and S1b–c). Considering that the yield of two-step chemical reaction is less than 100% (Figure 1b), the actual level of O-GlcNAc modification in cellular YTHDF1/3 should be even higher than 50%. Interestingly, although cell lysates of HEK293T and HeLa cells showed similar patterns and overall levels of O-GlcNAc modification (Figure 1f), the levels of endogenous O-GlcNAc on YTHDF1/3 in HeLa cells were much lower than those in HEK293T cells (Figure 1d–e). Nevertheless, three additional O-GlcNAcylated proteins^59–62^ showed similar modification levels in HEK293T and HeLa cells (Figure S1d–e). These results implied that O-GlcNAcylation of YTHDF1/3 is differentially regulated in a cell type-specific manner. Similarly, abundant O-GlcNAc modifications were observed in exogenously expressed YTHDF1/3 proteins but not YTHDF2 protein, and the modification levels were also different in HEK293T and HeLa cells (Figure 1g). Further analysis showed that the expression level of OGT in HEK293T is much higher than in HeLa whereas the expression level of OGA is similar (Figure 1h–i). The different expression pattern of modification enzymes may contribute to the different modification of YTHDF1/3 in these two cell lines. Together, these results suggest O-GlcNAcylation differences among the three YTHDF proteins, particularly, in the LCD of effector domains, could affect YTHDF protein functions in a cell-type specific manner.

### Dynamic O-GlcNAcylation on the low-complexity domain of YTHDF1/3

**Figure 2.**
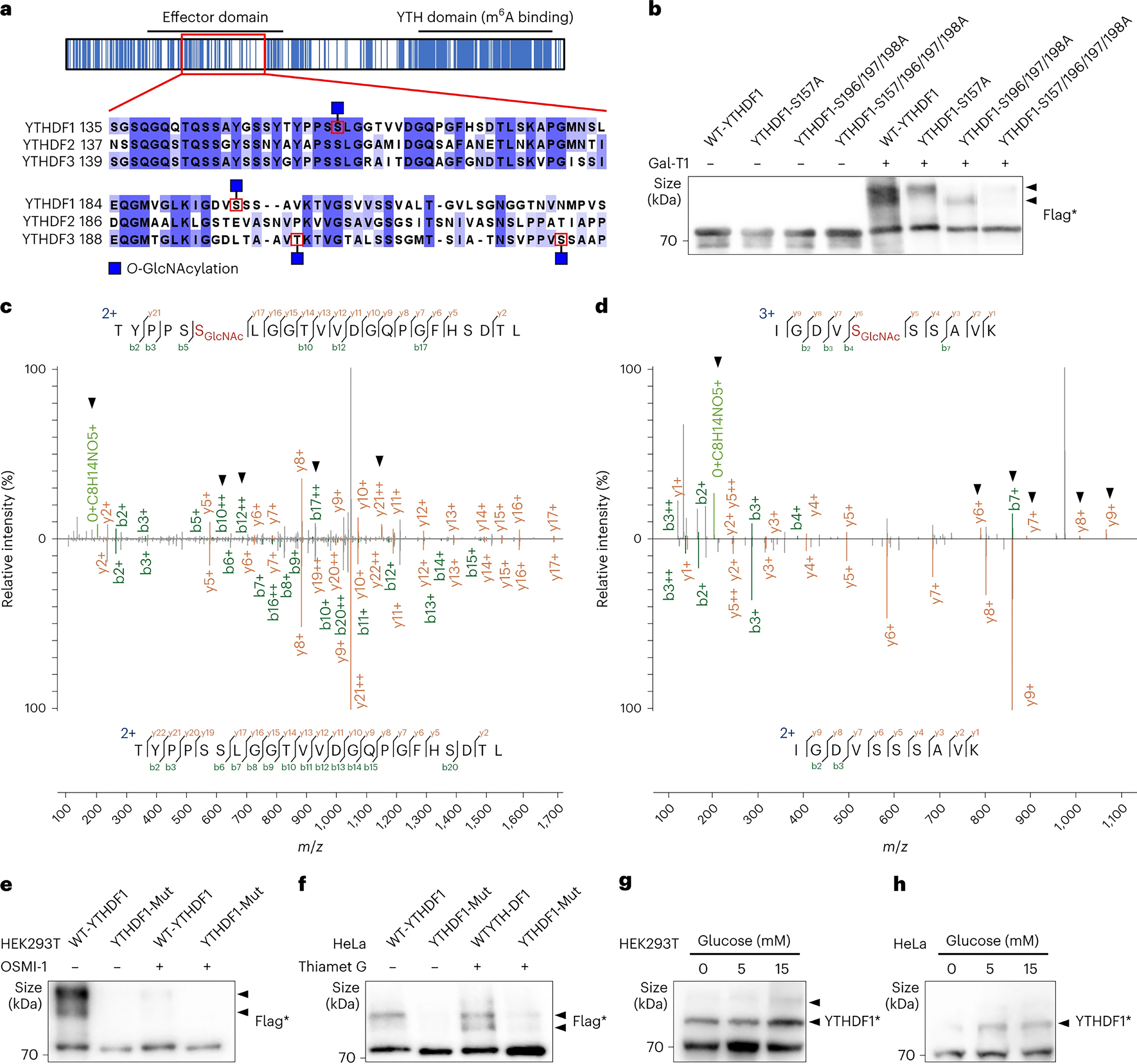
Reversible O-GlcNAcylation sites of YTHDF1/3. (a) The O-GlcNAc modified region in the effect domain is highlighted by a red rectangle with peptide sequence displayed. In the figure, blue indicates sequence conservation and the modified sites are highlighted by red boxes. (b) O-GlcNAcylation stoichiometry analysis of WT-YTHDF1 and YTHDF1-mutations. A representative mass spectrum of YTHDF1 peptide with S196 (c) or S157 (d) modified by O-GlcNAc. The spectra of unmodified peptides were used for side-by-side comparisons. The arrows pointed to the signature b/y-ions in modification spectrum. O-GlcNAcylation stoichiometry analysis of YTHDF1 in HEK293T treated with or without 50 μM OGT inhibitor OSMI-1 for 5 hr (e) or HeLa cells treated with or without 10 μM OGA inhibitor Thiamet G for 5 hr (f). O-GlcNAcylation stoichiometry analysis of YTHDF1 in HEK293T (g) or HeLa cells (h) with different glucose concentrations. Flag* and YTHDF1* denote samples which are performed O-GlcNAcylation stoichiometry analysis and immunoblotting by the respective antibodies. Arrowheads indicates the O-GlcNAc modified proteins.

We then sought to further investigate the modification sites and dynamics of O-GlcNAcylation on YTHDF1/3 (Figure 2a). The MS results detected two abundant O-GlcNAc modification sites on human YTHDF1 protein, at S157 and S196, respectively (Figure 2c–d). To verify these modification sites, the detected O-GlcNAc modification sites were mutated to alanine (Ala) that could not be modified by OGT, and changes in modification level of the mutant protein were quantified by the chemo-enzymatic assay (Figure 2b and S2a–d). The S157A mutation significantly decreased the overall level of O-GlcNAc on YTHDF1 (Figure 2b and S2a), while no significant changes were observed for the S196A mutant YTHDF1 (Figure S2b). Further mutagenesis experiments found compensatory increases of O-GlcNAc modification on nearby S197 or S198 upon S196A mutation (Figure S2c). We subsequently discovered that significant decreases in O-GlcNAc modification level could only be observed with all three Ser (S196–198) mutated to Ala (Figure 2b and S2d), and mutation of all four Ser (S157, S196–198) to Ala (YTHDF1-Mut) completely abolished O-GlcNAc modification on YTHDF1 (Figure 2b). Consistently, addition of the OGT inhibitor, OSMI-1, decreased the O-GlcNAc modification on YTHDF1, whereas addition of the OGA inhibitor, Thiamet G, increased the O-GlcNAc modification on YTHDF1 (Figure 2e–f) ^63–67^. These experiments testified that the O-GlcNAc modification on YTHDF1 was regulated by OGT and OGA. Lastly, the O-GlcNAc modification level on YTHDF1 changed significantly when HEK293T and HeLa cells were cultured in different glucose concentrations (Figure 2g–h), showing that the modification could respond to external nutrient changes.

Likewise, two O-GlcNAc modification sites, T205 and S229, were identified on YTHDF3, respectively (Figure S2e–g). Notably, YTHDF1/3 had very different modification sites and conservation of their surrounding peptide sequence (Figure 2a). Similarly, the O-GlcNAcylation modification on YTHDF3 could also be dynamically regulated (Figure S2h).

### O-GlcNAcylation of YTHDF1/3 impairs their association with translation machineries

**Figure 3.**
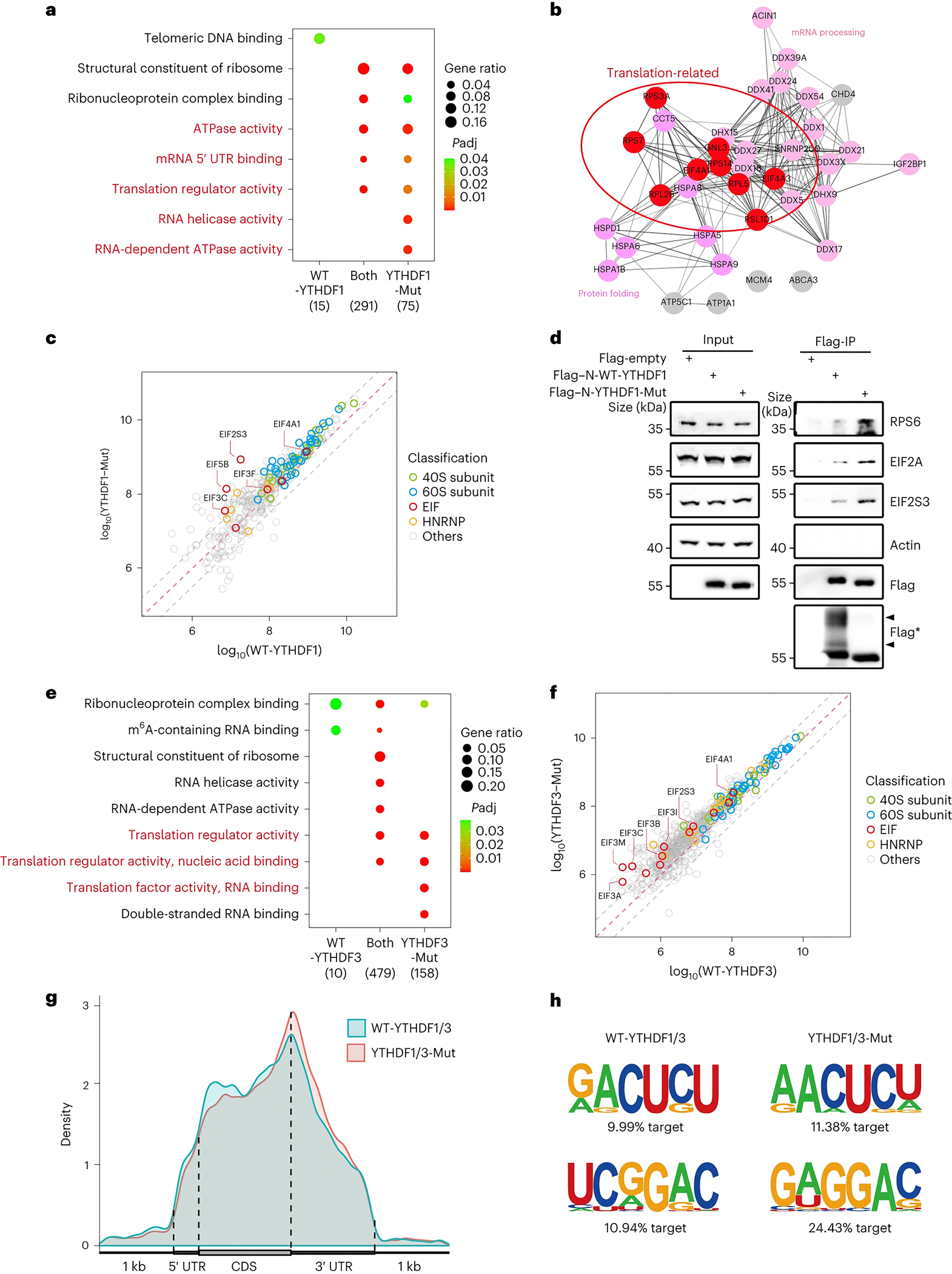
O-GlcNAcylation of YTHDF1/3 repressed their protein interaction. (a) Enrichment analysis of proteins enriched in WT-YTHDF1, YTHDF1-Mut and Both groups by immunoprecipitation experiments and label-free quantification with Flag tagged WT-YTHDF1 and YTHDF1-Mut in HEK293T cells. The numbers of enriched proteins are annotated at the bottom. Size of circle denotes gene ratio. Color of circle denotes adjusted p value. (b) Interaction network of enrichment groups highlighted by red in (a) and exclusively enriched in the YTHDF1-Mut group. Proteins involved in translation, protein folding and mRNA processing are annotated by the color. The translation-related proteins are highlighted by a red oval. (c) Pairwise comparison of label-free quantification of co-immunoprecipitated proteins interacting with WT-YTHDF1 and YTHDF1-Mut from HEK293T cells. And the position of the x and y axes reflected the average LFQ intensities in the WT-YTHDF1 and YTHDF1-Mut, respectively. YTHDF1 protein partners were plotted in green (40S ribosomal proteins), blue (60S ribosomal proteins), red (translation initiation factors, EIF), and orange (Heterogeneous nuclear ribonucleoprotein, HNRNP), respectively. EIFs enriched in YTHDF1-Mut group were annotated. The diagonals denoted the fold change threshold of −0.5, 0 and 0.5 (Log_10_(WT-YTHDF1 / YTHDF1-Mut)). (d) Co-IP analysis of RPS6, EIF2A and EIF2S3 by N-WT-YTHDF1 and N-YTHDF1-Mut from HEK293T cells. Flag* denotes samples which are performed O-GlcNAcylation stoichiometry analysis and immunoblotting by flag antibodies. Arrowheads indicates the O-GlcNAc modified proteins. (e) Enrichment analysis of proteins enriched in WT-YTHDF3, YTHDF3-Mut and Both groups by immunoprecipitation experiments and label-free quantification with Flag tagged WT-YTHDF3 and YTHDF3-Mut in HEK293T cells. (f) Pairwise comparison of label-free quantification of co-immunoprecipitated proteins interacting with WT-YTHDF3 and YTHDF3-Mut from HEK293T cells. The diagonals denoted the fold change threshold of −0.5, 0 and 0.5 (Log_10_(WT-YTHDF3 / YTHDF3-Mut)). (g) Distribution of PAR-CLIP peaks across the length of mRNA. (h) Binding motif of WT-YTHDF1/3 (left) and YTHDF1/3-Mut (right) from PAR-CLIP of HEK293T cells.

YTHDF proteins exert their biological functions through recruiting interacting protein complexes using their N-terminal effector domains ^33, 34, 68, 69^. The effector domains can form biomolecular condensates through hydrophobic interactions, which contribute to specific protein interactions and phase separation ^70–72^. Interestingly, all the hydrophilic O-GlcNAc modifications were located in the LCD. Therefore, we hypothesized that the hydrophilic O-GlcNAc modification in the hydrophobic LCD of YTHDF1/3 may affect their participation in RNA-protein granules. To test this hypothesis, we mapped protein interaction networks of O-GlcNAc-modified YTHDF1/3 (WT-YTHDF1 and WT-YTHDF3) and unmodified YTHDF1/3 (YTHDF1-Mut and YTHDF3-Mut), respectively in HEK293T cells. Immunoprecipitation experiment showed that 381 proteins were detected as potential interacting proteins of YTHDF1 (Figure 3a and Data S2). Most (291 out of 381) were found in both the WT-YTHDF1 group and the YTHDF1-Mut group. Consistent with the identified functions of YTHDF1 ^33, 34, 68, 69^, these proteins are enriched in biological functions including mRNA binding, translation regulation, and RNA processing. Interestingly, many of the interacting proteins (75 out of 381) were found exclusively in the YTHDF1-Mut group (Figure 3a), in line with the hypothesis that the O-GlcNAc modification of YTHDF1 might suppress YTHDF1 binding to these proteins. Among them, translation regulators were enriched in these 75 proteins (Figure 3b). Subsequent pairwise analysis showed that many translation-associated proteins were enriched in the YTHDF1-mut group, in contrast, no translation-associated proteins were enriched in the WT-YTHDF1 group (Figure 3a, c). RPS6, EIF2A, and EIF2S3 were detected in the YTHDF1 pull-down fractions, validating the results of the MS analysis (Figure 3d). In addition, *in vitro* pull-down experiments confirmed that YTHDF1/3 directly interacts with several EIF proteins, such as EIF2S3, EIF3M, and EIF4E, through their LCD domains (Figure S3a–d). Together, these data suggested that unmodified YTHDF1 binds translation-associated proteins and that the O-GlcNAc modification mainly inhibits the binding of YTHDF1 to translation-associated proteins with limited effects on other interacting proteins (Figure 3a–c). Note that the effector domain alone could be efficiently O-GlcNAcylated (Figure 3d), suggesting that this modification process was independent of their m^6^A binding. Similar conclusions were obtained in the comparison of WT-YTHDF3 and YTHDF3-Mut protein interaction networks (Figure 3e–f and Data S2).

We next investigated effects of YTHDF1/3 O-GlcNAcylation on mRNA binding with cross-linking immunoprecipitation followed by high-throughput sequencing (CLIP-seq). WT-YTHDF1 and YTHDF1-Mut exhibited similar m^6^A binding profiles on mRNA, which were enriched near the stop codons and in the 3’-UTRs (Figure 3g and Data S3). Electrophoretic mobility shift assay (EMSA) experiments Further confirmed that O-GlcNAcylated YTHDF1/3 could bind to the m^6^A RNA probe with similar binding affinity as unmodified YTHDF1/3 (Figure S3e–h). Thus, O-GlcNAc modification on the effector domains does not notably change substrate binding activity of the m^6^A binding domain (Figure 3g–h and Data S3). Taken together, these experiments indicated that the O-GlcNAc modification on YTHDF1/3 LCDs can reach high levels in certain cells to influence the recruitment of protein translation regulators and potentially impact the function of YTHDF1 and YTHDF3.

### O-GlcNAcylation on YTHDF1/3 suppresses mRNA translation promotion

**Figure 4.**
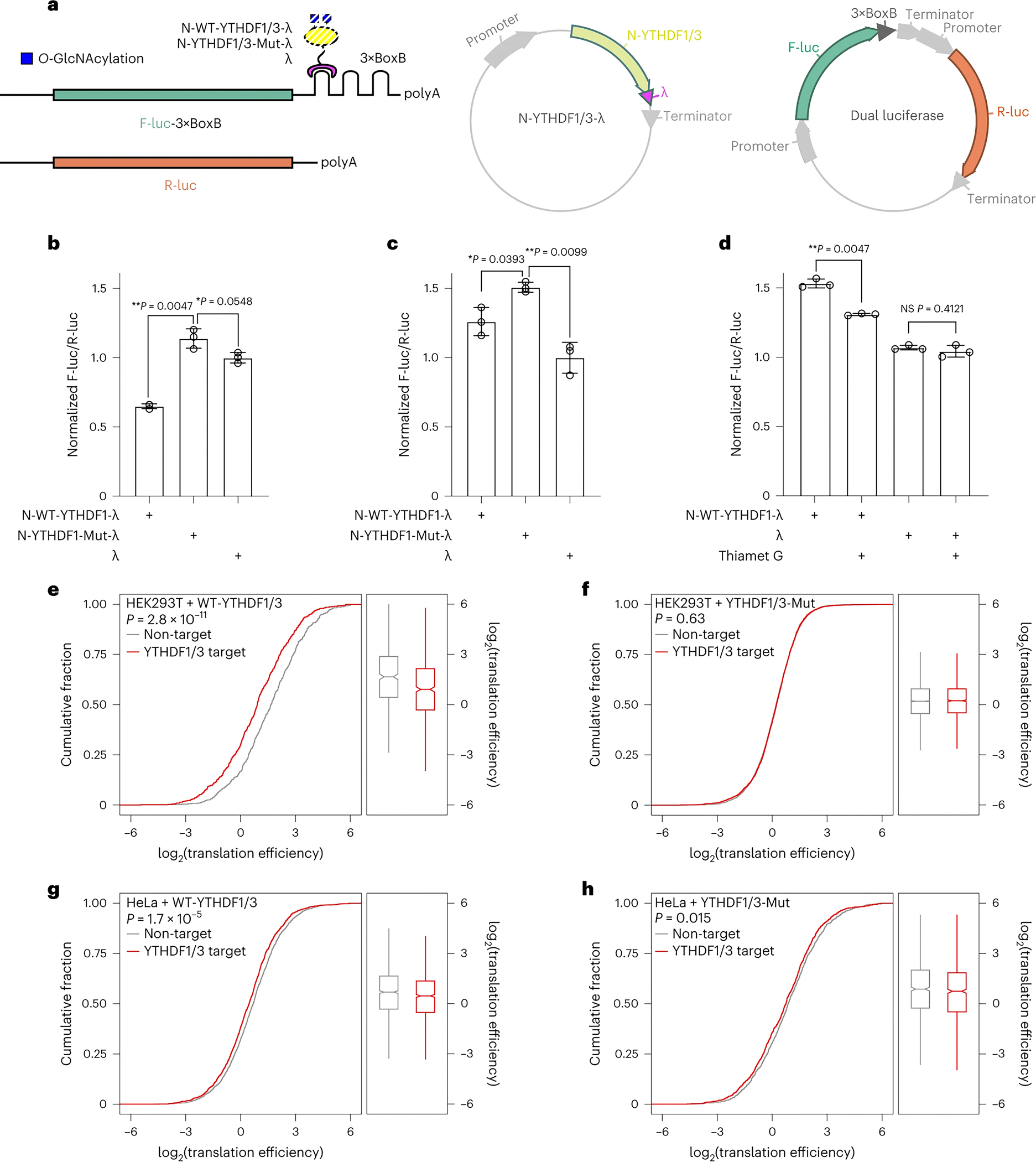
O-GlcNAcylation of YTHDF1 repressed target mRNA translation efficiency. (a) Schematic of the tethering reporter assay. 3×BoxB sequence is inserted at 3’ UTR of firefly luciferase mRNA reporter (F-luc-3BoxB). The N-terminal domain of WT-YTHDF1/3 and YTHDF1/3-Mut are fused with λ peptide used to recognize BoxB RNA sequence. The expression of luciferase mRNAs is examined in HEK293T and HeLa cells, and the construct containing λ peptide alone is used as a negative control. The F-luc signal is normalized by R-luc in the same plasmid. Translation efficiency analysis of N-WT-YTHDF1-λ, N-YTHDF1-Mut-λ and λ in HEK293T (b) or HeLa cells (c). Error bar, mean ± SD, n = 3 biologically independent repeats. Significance was calculated using two-sided t-test. (d) Translation efficiency analysis of N-WT-YTHDF1-λ and λ treated with or without OGA inhibitor Thiamet G in HeLa. Thiamet G: 5 μM, 5 hr. Error bars, mean ± SD, n = 3 biologically independent repeats. Significance is calculated using two-sided t-test. (e-h) Cumulative distribution of translation efficiency for YTHDF1/3 target mRNA and non-target mRNA. *Ythdf1/3* knock down HEK293T (e-f) and HeLa (g-h) are rescued by WT-YTHDF1/3 (e, g) or YTHDF1/3-Mut (f, h). Distribution with boxplot bounds depict quartile 1, median and quartile 3, with whiskers at 1.5× interquartile range and outlier points. The p values were calculated using two-sided t-test (right boxes).

Because O-GlcNAcylation on YTHDF1/3 affects their interaction with proteins involved in translation, we examined whether translation is affected or not by using a previously reported luciferase-based tethering reporter assay (Figure 4a) ^33^. The results showed that the translation efficiency of YTHDF1-Mut-λ was increased by ~50% in HeLa cells and ~20% in HEK293T cells, respectively, indicating that the binding of unmodified YTHDF1 (YTHDF1-Mut) to the translation components was sufficient to promote mRNA translation (Figure 4b–c and S4a–c). In contrast, the increase in translation efficiency was much lower in HeLa cells and even decreased in HEK293T cells when O-GlcNAc modified YTHDF1 (WT-YTHDF1) was used (Figure 4b–c and S4a–c). Indeed, immunofluorescence assay revealed a colocalization of YTHDF1/3 with m^6^A RNA in P-bodies (Figure S5), suggesting that YTHDF1/3 may also promote m^6^A mRNA degradation, but in an O-GlcNAcylation-independent manner (Figure S4h). A recent study suggests that this destabilization effect is m^6^A independent ^38, 73^. The mRNA degradation effect contributed to the reduced translation efficiency of the WT-YTHDF1 group compared to the λ control group (Figure 4b).

Because YTHDF1 carries a much higher level of O-GlcNAc modification in HEK293T cells than that in HeLa cells, we reasoned that the O-GlcNAc modification of YTHDF1 suppresses its translation-promoting function and this effect is dependent on the O-GlcNAc modification stoichiometry. To test this hypothesis, the OGT or OGA inhibitor was added to further decrease or increase the O-GlcNAc modification level on YTHDF1 (Figure 4d and S4d–e), respectively. The translation promotion effect of YTHDF1 was attenuated by OGA inhibitor treatment in HeLa cells (Figure 4d and S4e), whereas the translation efficiency of YTHDF1 was enhanced by low concentrations of OGT inhibitor in HEK293T cells (Figure S4d). These results testified that the O-GlcNAc modification of YTHDF1 had a negative effect on its ability to promote mRNA translation. Similar impacts for the effector domain of YTHDF3 variants were also observed (Figure S4f–i). Moreover, the expression level of YTHDF1/3 did not show any significant changes upon inhibitor treatments (Figure S6a–d). The cellular localization of YTHDF1/3 was not visibly affected by O-GlcNAc modification in both HeLa and HEK293T cells (Figure S6e–f). Together, these data support a model that the binding of YTHDF1 and YTHDF3 to the translation components promotes mRNA translation, and this binding is negatively regulated by O-GlcNAc modification on their effector domains (Figure 4b–d, S4c–j).

To confirm the suppression of translation-promoting function of YTHDF1/3 by O-GlcNAcylation transcriptome-wide, ribosome profiling analysis was applied (Figure S7a–c and Data S4). In *Ythdf1/3* knock down HEK293T cells, a notable decrease in translation of m^6^A-modified mRNAs (YTHDF1/3 targets) was detected in contrast to unmodified mRNAs (non-target) when rescued with WT-YTHDF1 and WT-YTHDF3 (Figure 4e and S7d); note that these WT-YTHDF1 and WT-YTHDF3 are heavily O-GlcNAc modified. In comparison, the translation of YTHDF1/3 target mRNAs and unmodified mRNAs was unchanged when rescued with YTHDF1-Mut and YTHDF3-Mut (cannot be O-GlcNAc modified), supporting the notion that O-GlcNAcylation on YTHDF1 and YTHDF3 inhibits their translation promotion functions (Figure 4f and S7e). Similarly, WT-YTHDF1 and WT-YTHDF3 restoration repressed the translation of YTHDF1/3 target mRNAs in HeLa cells compared to YTHDF1-Mut and YTHDF3-Mut (Figure 4g–h and S7f–g). Notably, a weaker reduction in translation by WT-YTHDF1 and WT-YTHDF3 was observed in HeLa cells compared to HEK293T cells, in parallel with lower O-GlcNAc modification level of YTHDF1 in HeLa cells. Additionally, the tethering reporter assay showed that high glucose concentration down-regulated the translation of substrate mRNA (Figure S7h), which is most likely due to the altered O-GlcNAcylation of YTHDF1/3 (Figure 2g–h). Ribosome profiling analysis of HeLa cells with or without glucose treatment further supported this effect on target transcripts (Data S5). Although the overall translation efficiency of mRNAs increased with high glucose treatment (Figure S7i), the translation efficiency of YTHDF1 targeting mRNAs ^33^ was down-regulated with high glucose treatment (Figure S7j) in a m^6^A number-dependent manner ^35^ (Figure S7k).

To summarize this part, all these results are consistent with previous observations showing translation promotion function of the wild-type YTHDF1 in HeLa cells but not in HEK293T cells. The cell-type specific translation promotion function of YTHDF1 is at least partially regulated through its O-GlcNAcylation. The high O-GlcNAcylation of YTHDF1 in HEK293T cells shields the protein from interacting with translation components, which suppresses its translation-promotion function. In HeLa cells, YTHDF1 protein level is high and its O-GlcNAcylation is low, leading to more YTHDF1 interacting with ribosome components for translation promotion of m^6^A-methylated mRNAs.

### Cell cycle-specific dynamic O-GlcNAcylation of YTHDF1/3 regulates mRNA translation

**Figure 5.**
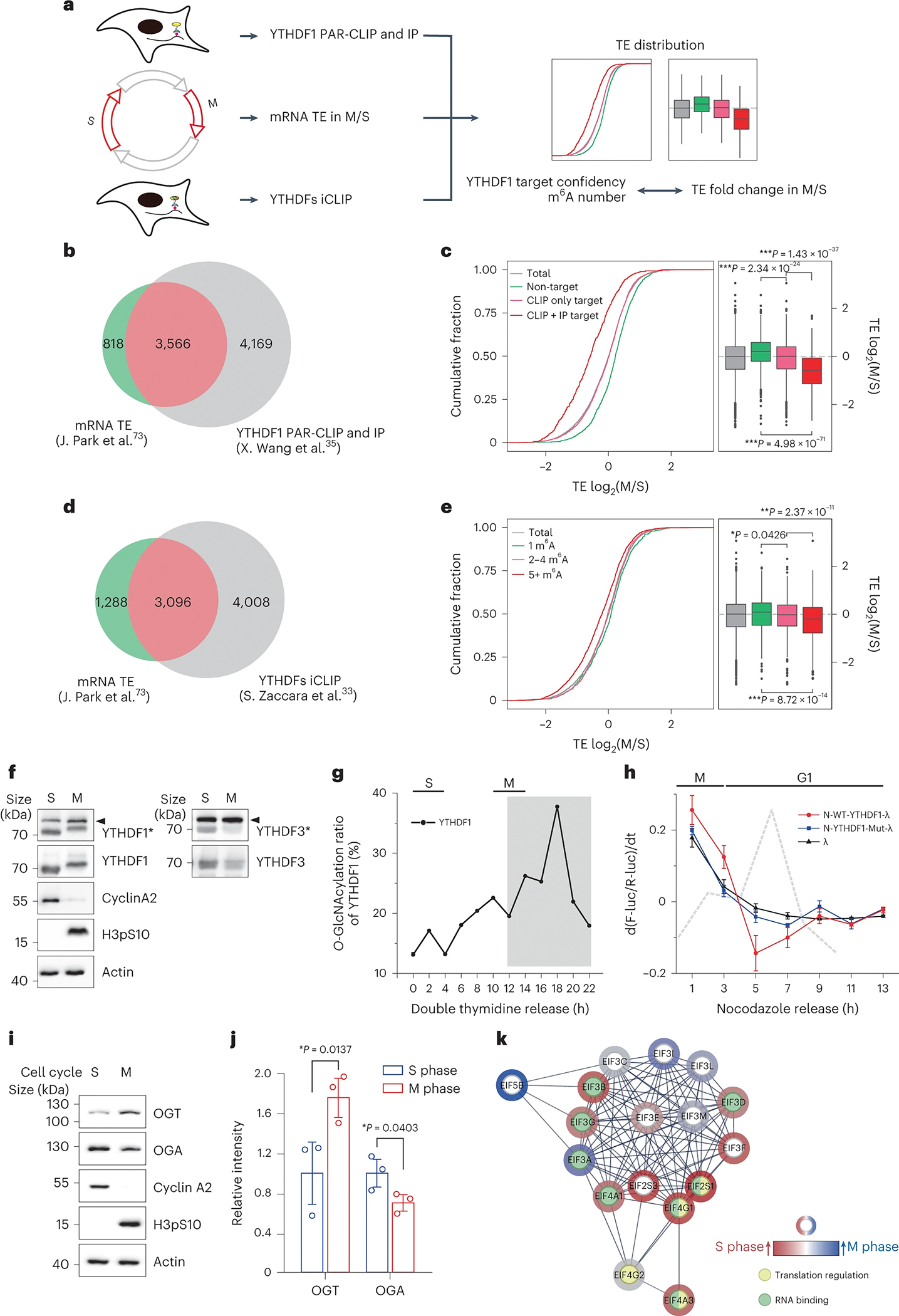
Dynamic O-GlcNAcylation of YTHDF1/3 regulated m^6^A mRNA translation efficiency in a cell cycle dependent manner. (a) Strategy of dataset analysis for m^6^A containing mRNA translation efficiency in M and S phase. The mRNA translation efficiency, YTHDF1 PAR-CLIP and IP, and YTHDFs iCLIP datasets are acquired and combined. Distribution of mRNA translation efficiency in different groups is presented and compared. (b) Overlap of mRNAs of translation efficiency dataset and YTHDF1 target mRNAs dataset. The dataset of m^6^A-modified mRNA in HeLa cells is generated by PAR-CLIP and/or the immuno-purified ribonucleoprotein complex of YTHDF1. The dataset of the translation efficiency of mRNA in HeLa cells is generated by ribosome profiling in the M and S phases, respectively. The mRNAs in green and red are used for subjected analysis. (c) Cumulative distribution log_2_-fold changes of translation efficiency of YTHDF1 target mRNAs between M and S phase (left box). Distribution with boxplot and p values are calculated using two-sided t-test (right box). Boxplot bounds depict quartile 1, median and quartile 3, with whiskers at 1.5× interquartile range and outlier points. (d) Overlap of mRNAs of translation efficiency dataset and YTHDFs iCLIP for m^6^A site numbers dataset. mRNAs in green and red are used for subjected analysis. (e) Cumulative distribution log_2_-fold changes of translation efficiency of mRNAs containing different m^6^A numbers between M and S phase (left box). The dataset of m^6^A-modified mRNA in HeLa cells is generated by iCLIP. Distribution with boxplot and p values are calculated using two-sided t-test (right box). Boxplot bounds depict quartile 1, median and quartile 3, with whiskers at 1.5× interquartile range and outlier points. (f) O-GlcNAcylation stoichiometry analysis for YTHDF1/3 in HeLa at S and M phase. Cyclin A2 and H3pS10 are used as cell cycle marker of S and M phase. YTHDF1* and YTHDF3* denote samples which were performed O-GlcNAcylation stoichiometry analysis and immunoblotting by respective antibodies. Arrowheads indicates the O-GlcNAc modified proteins. (g) Quantification analysis of O-GlcNAcylation stoichiometry analysis for YTHDF1 in HeLa at different cell cycle stages by double thymidine release. Grey shading indicates the dynamic change window shown in (h). (h) Monitor of luciferase expression in HeLa at the indicated cell cycle stages by nocodazole release. Y axis, d(F-luc/R-luc)/dt, indicating the changing rate of protein production. Error bar, mean ± SD, n = 3 biologically independent repeats. (i) Expression level comparison of OGT and OGA between S and M phase in HeLa by western blot. Cyclin A2 and H3pS10 were used as cell cycle marker of S and M phase. Actin was used as loading control. (j) Quantification analysis of expression level of OGT and OGA in (i). Error bar, mean ± SD, n = 3 biologically independent repeats. (k) Interacting protein analysis of flag-YTHDF1 between S and M phase in HeLa by immunoprecipitation and LC-MS/MS. The EIFs were presented, and the outer layer of the circle was colored based on the fold change of label-free quantification. The proteins involved in translation regulation and RNA-binding were colored in inner layer of the circle by yellow and green respectively.

Next, we asked whether this O-GlcNAcylation-regulated mRNA translation is physiologically relevant. The cellular mRNA translation is regulated in a cell cycle-dependent manner ^74^, although, whether m^6^A is implicated or not is unknown. To this end, we quantified the translation efficiency of m^6^A-modified mRNAs in the S and M phases in HeLa cells using a published dataset (Figure 5a, S8a and Data S6) ^33, 35, 74^. 3,566 target mRNAs of YTHDF1 (PAR-CLIP target mRNAs) were actively translated in both S and M phases (Figure 5b and Data S6). Comparing the translation efficiency of these target mRNAs between M and S phases showed a significant decrease upon entering M phase, using non-target mRNAs (unmodified mRNAs) for normalization. In addition, YTHDF1-enriched PAR-CLIP target mRNAs (PAR-CLIP+IP) showed even lower translation efficiency than PAR-CLIP alone target mRNAs (Figure 5c). These analyses suggested that the translation of YTHDF1 target mRNAs is regulated on a cell cycle stage-specific manner. To further study this phenomenon, we compared the translation efficiency of mRNAs carrying different numbers of m^6^A modification sites between M and S phases. The results revealed that the translation efficiency (M phase versus S phase) gradually decreased as the number of m^6^A modifications on mRNA increased (Figure 5d–e, S8b and Data S6). Therefore, the translation efficiency of m^6^A-modified mRNA is lower in the M phase than in S phase.

We hypothesized the dynamic O-GlcNAc modification of YTHDF1/3 may play an important role in regulating translation efficiency during cell cycle^75, 76^. Western blot analysis indeed showed that the level of YTHDF1 O-GlcNAc modification was significantly increased in M phase (Figure 5f). Further analysis of the O-GlcNAc modification of YTHDF1 revealed a cell cycle-dependent dynamic (Figure 5g and S8c). In addition, luciferase-based tethering reporter assay suggested that O-GlcNAc modification level of N-WT-YTHDF1-tethered group was negatively correlated with the translation efficiency of its target mRNA (Figure 5h and S8d). This correlation was dependent on the O-GlcNAc modification of the protein, which was not observed in the N-YTHDF1-Mut-tethered group (Figure 5h and S8d). Furthermore, the expression level of OGT is much higher in M phase than in S phase in HeLa cells, whereas the expression level of OGA is much lower (Figure 5i–j). The differential expression pattern of modification enzymes may contribute to the differential modification of YTHDF1/3 through cell cycle; YTHDF1/3 showed similar spatial localization at different cell cycle stages (Figure S8f). Moreover, the IP-MS of YTHDF1 in M and S phase revealed that YTHDF1 appears to recruit more translation initiation factors in S phase (with low O-GlcNAcylation) when compared to M phase (with high O-GlcNAcylation) (Figure 5k). Therefore, we conclude that dynamic O-GlcNAc modification may regulate the translation efficiency of m^6^A-modified mRNA during the cell cycle.

### O-GlcNAcylation of YTHDF1/3 regulates the assembly, stability, and disassembly of stress granule

**Figure 6.**
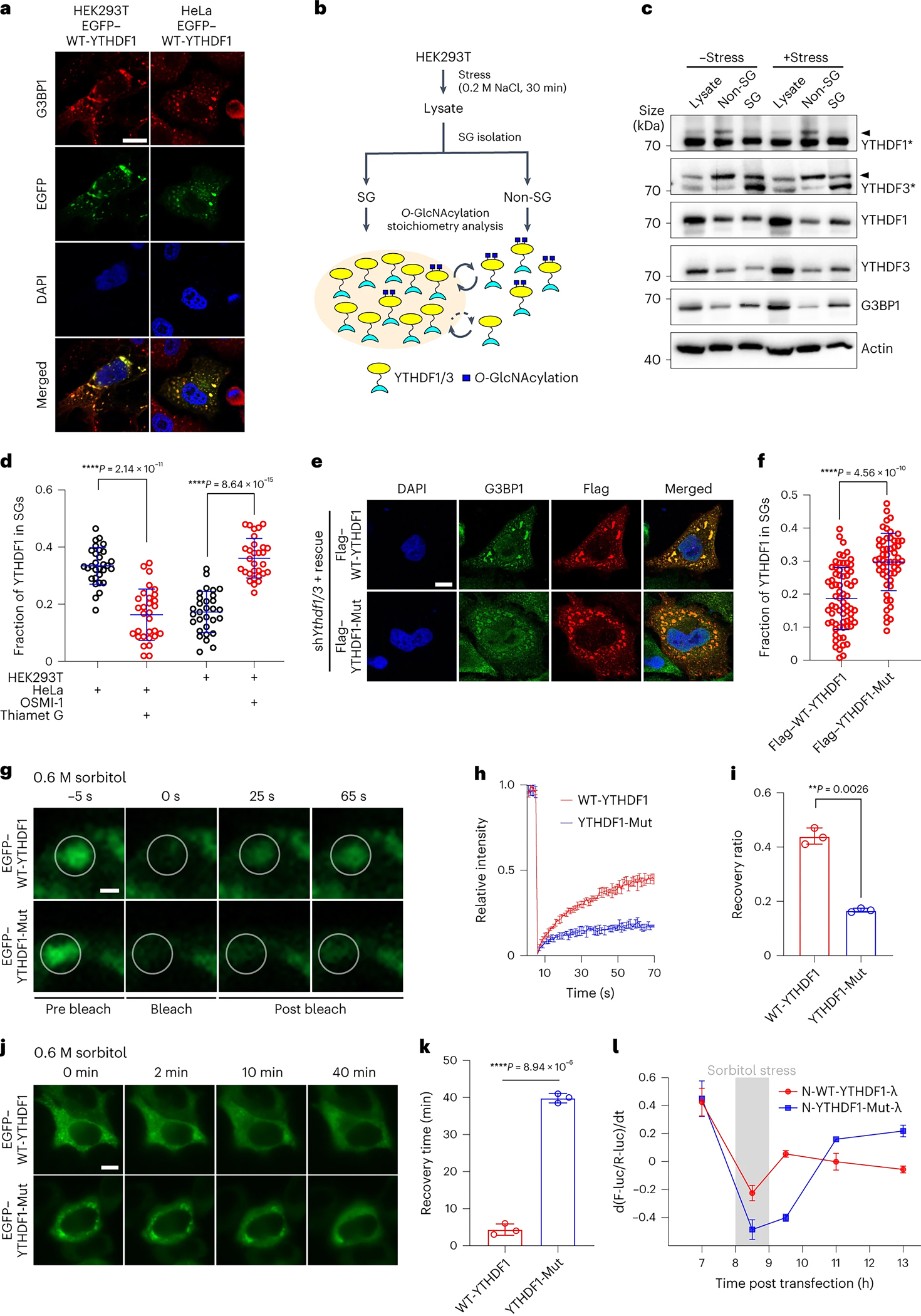
O-GlcNAcylation of YTHDF1/3 increased their dynamic nature in stress granules. (a) Colocalization of EGFP-YTHDF1 and G3BP1 under stress in HEK293T and HeLa. Scale bars: 10 μm. (b) Strategy of O-GlcNAcylation stoichiometry analysis inside or outside of stress granules. HEK293T cells are treated with NaCl stress. Then, the lysate is centrifuged for SGs isolation and subjected to O-GlcNAcylation stoichiometry analysis for YTHDF1/3. (c) O-GlcNAcylation stoichiometry analysis of YTHDF1/3 in HEK293T. G3BP1 is used as stress granules marker. SG, stress granule fraction. non-SG, lysate without stress granule fraction. YTHDF1* and YTHDF3* denote samples were performed O-GlcNAcylation stoichiometry analysis and immunoblotting by respective antibodies. Arrowheads indicates the O-GlcNAc modified proteins. (d) Quantification result of the fraction of YTHDF1 in stress granules, in HEK293T overexpressing EGFP-WT-YTHDF1 treated with or without OSMI-1, or in HeLa overexpressing EGFP-WT-YTHDF1 treated with or without Thiamet G, n = 30. Representative immunostaining result is shown in Fig S9a–b. Error bar, mean ± SD. Significance was calculated using two-sided t-test. (e) Immunostaining of *Ythdf1/3* knock down HeLa rescued by Flag-WT-YTHDF1 or Flag-YTHDF1-Mut and treated with 0.6 M sorbitol stress. Scale bars: 10 μm. (f) Quantification result of fraction of YTHDF1 in stress granules of (e), n = 69, 55. Error bar, mean ± SD. Significance is calculated using two-sided t-test. (g-i) FRAP analysis of EGFP-WT-YTHDF1 and EGFP-YTHDF1-Mut treated with 0.6 M sorbitol stress in HeLa. Scale bars: 1 μm. Error bar, mean ± SD, n = 3. Significance was calculated using two-sided t-test. (j-k) Time lapse imaging of EGFP-WT-YTHDF1 and EGFP-YTHDF1-Mut released from 0.6 M sorbitol stress in living HeLa cells. Scale bars: 10 μm. Error bar, mean ± SD, n=3. Significance is calculated using two-sided t-test. (l) Monitor of luciferase expression in HEK293T after 1 hr sorbitol stress treatment. Grey shading indicates the sorbitol treatment window. Y axis, d(F-luc/R-luc)/dt, indicating the changing rate of protein production. Error bar, mean ± SD, n = 3 biologically independent repeats.

Protein O-GlcNAcylation is known to disfavour condensate formation ^77–80^. Recent studies have also shown that m^6^A-modified mRNAs undergo liquid-liquid phase separation (LLPS) with YTHDF proteins, leading to the formation of endogenous LLPS compartments, such as stress granules, for compartment-specific mRNA regulation ^70–72, 81^. Although all three YTHDF proteins were reported to associate with LLPS in stress granules, YTHDF1/3, but not YTHDF2, were found to promote stress granule formation ^72^. We postulated that O-GlcNAcylation on YTHDFs could play a role in stress granule formation. We first confirmed that YTHDF1/3 were enriched in stress granule in response to chemical inducer of stress. Sorbitol or NaCl were used as chemical inducers in this research because these two osmotic stress inducers have been widely used and can induce robust stress on various cell lines ^82, 83^. The results showed that EFGP-tagged YTHDF1 co-localized well with G3BP1, a stress granule biomarker, in both HEK293T and HeLa cells stressed with sorbitol (Figure 6a) ^84, 85^.

To further investigate the relationship between YTHDF1/3 O-GlcNAc modification and stress granule dynamics, we separated stress granule components from HEK293T whole cell lysate and examined the existence of O-GlcNAc-modified YTHDF proteins in the stress granules by western blotting (Figure 6b). We specifically detected O-GlcNAc-modified YTHDF1/3 outside of the stress granules (non-SG) rather than inside of the stress granules (SG), regardless of whether the stress granule formation was induced by chemical treatment or not (Figure 6c), consistent with the role of O-GlcNAcylation disrupting condensate formation. Furthermore, immunostaining showed that inhibition of the O-GlcNAc modification on YTHDF1 through mutation or addition of OGT inhibitors in HEK293T resulted in a notable increase in the number of stress granules per cell and the fraction of YTHDF1 in stress granules (Figure 6d and S9a, c). Similarly, inhibition of O-GlcNAc modification on YTHDF1 through mutation increased the number of stress granules per cell in HeLa cells as well (Figure S9b, d), while promoting O-GlcNAc modification on YTHDF1 by adding OGA inhibitor had the opposite effect (Figure 6d and S9b, d). In addition, we confirmed that silencing of *Ythdf1/3* attenuated the formation of stress granule (Figure S9g–i) ^72^, whereas rescue with YTHDF1-Mut enhanced assembly of YTHDF1-containing stress granule compared to WT-YTHDF1 (Figure 6e–f, S9j–k). These results demonstrated that O-GlcNAc modification of YTHDF1 inhibits the assembly of YTHDF1-containing stress granule.

Considering that hydrophobic interaction plays an important role in stabilizing LLPS, the hydrophilic nature of O-GlcNAc modification reduces the stability of stress granules, resulting in a more dynamic property of LLPS (Figure 6b). We performed photo bleaching experiments to probe the dynamic nature of NaCl or sorbitol-induced LLPS with the expression of EGFP-tagged unmodified YTHDF1 (YTHDF1-Mut) and modified YTHDF1 (WT-YTHDF1) (Figure 6g–i and S9l–n). A rapid recovery of EGFP fluorescence was observed in LLPS expressing WT-YTHDF1 even under high concentrations of different stress granule-stimulating compounds, in comparison to the YTHDF1-Mut group (Figure 6g–i, S9l–n and S10a–e), indicating a critical role of O-GlcNAcylation on condensate dynamics. The time-lapse imaging experiments revealed that O-GlcNAc-modified YTHDF1 exhibited a much more rapid disassembly process of YTHDF1-containing stress granule compared to unmodified YTHDF1 (Figure 6j–k and S10f–g), further indicating the O-GlcNAcylation increase trafficking dynamics of YTHDF1 in and out of granules. We next investigated the translation promotion function of YTHDF1 during stress response and recovery stage. In this context, Luciferase-based tethering reporter assay were performed on cells with 1 hour sorbitol treatment. Compared to N-YTHDF1-Mut group, the target mRNA translation in the N-WT-YTHDF1 group showed a rapid restoration in the early recovery stage (Figure 6l). The accelerated translation restoration was likely due to the increased trafficking dynamics of O-GlcNAc-modified YTHDF1 in the granules (Figure 6l). Indeed, an increase O-GlcNAcylation of YTHDF1 was detected during the early stress recovery stage (Figure S9o–p). To further investigate the molecular mechanism, the *in vitro* droplet assay was performed with purified O-GlcNAcylated and unmodified YTHDF1/3. The results showed that O-GlcNAcylation decreased the formation of YTHDF1/3 protein condensates and increased the dynamic of YTHDF1/3 condensates (Figure S11a-i). In addition, we have also carried out *in vitro* droplet assay with purified G3BP1 and m^6^A RNA probe to strengthen the effect of YTHDF1 O-GlcNAcylation in regulating the formation and dynamics of SG LLPS (Figure S11j-o). Our results showed that O-GlcNAc modification of YTHDF1/3 has an important function in regulating the life cycle of stress granules, including their assembly and disassembly, by promoting the dynamics of YTHDF1/3 in stress granules for better stress recovery.

## Discussion

**Figure 7.**
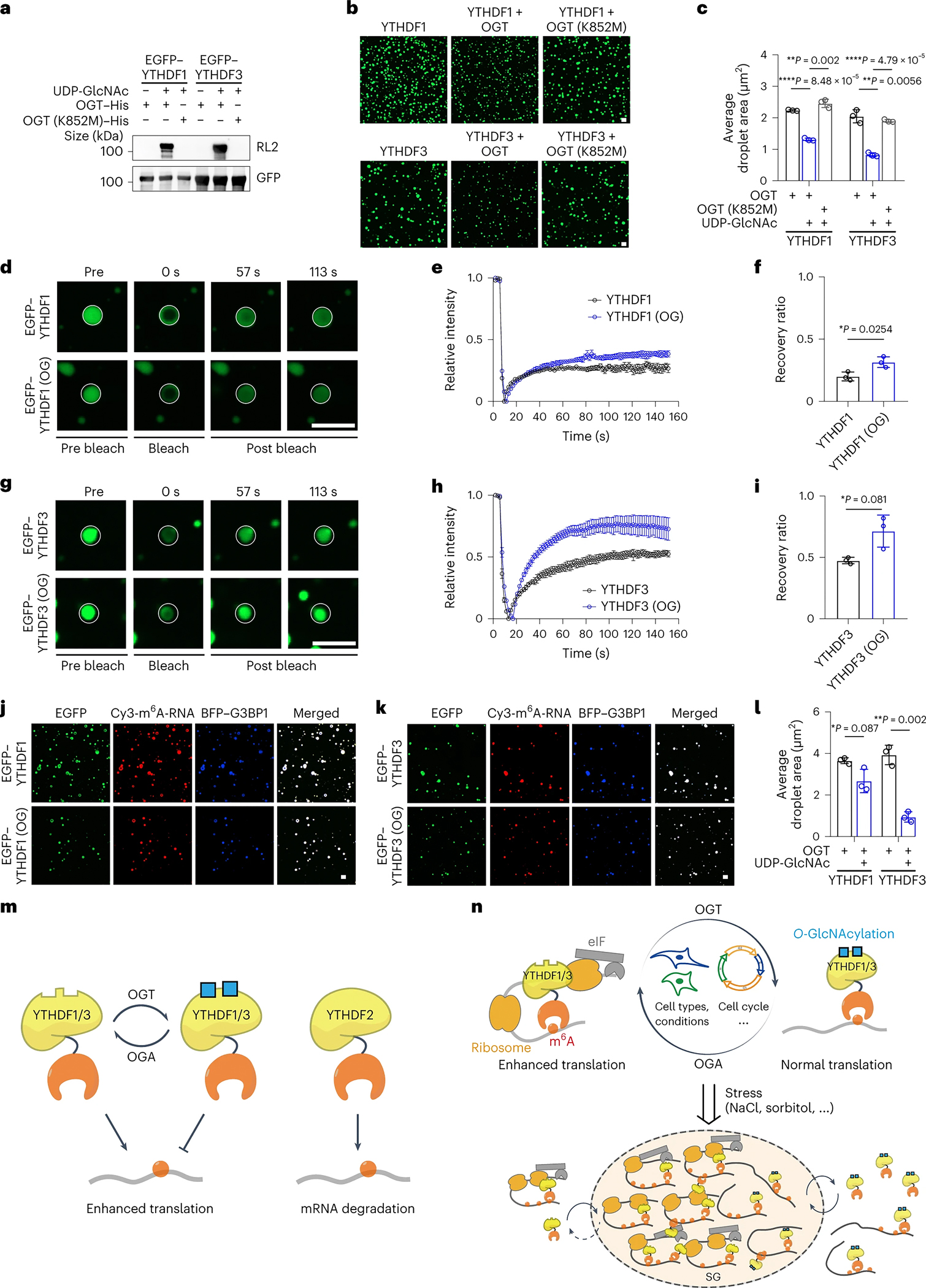
A proposed model of regulation of YTHDF1/3 by O-GlcNAcylation. (a) YTHDF1/3 instead of YTHDF2 can be reversibly modified by protein O-GlcNAcylation. YTHDF1/3 mediates enhanced translation of m^6^A-modified mRNA. However, the enhanced translation is suppressed by O-GlcNAcylation. In contrast, YTHDF2 reduces the stability of m^6^A-modified mRNA. (b) O-GlcNAcylation of YTHDF1/3 is reversibly regulated in different cell lines, culture conditions and cell cycle state. High O-GlcNAcylation of YTHDF1/3 represses m^6^A-modified mRNA translation promotion. Meanwhile, low O-GlcNAcylation of YTHDF1/3 promotes m^6^A-modified mRNA translation by recruiting eIF, ribosome and other translation related proteins. O-GlcNAcylation of YTHDF1/3 regulate the assembly, stability, and disassembly of stress granule for a better recovery from stress.

The cytosolic m^6^A readers YTHDF1, YTHDF2 and YTHDF3 proteins control the cytoplasmic fate of a large portion of m^6^A-modified mRNAs and play critical roles in m^6^A biology. Despite extensive past research on the YTHDF proteins, the cellular functions and underlying regulatory mechanisms have been confusing from different reports. Whether YTHDF proteins act in similar or distinct manners, and why YTHDF1/3 proteins exhibit distinct effects in different cell lines and experimental conditions are two key questions. For instance, YTHDF1/3 was reported to promote translation efficiency of target transcripts in HeLa and HEC-1-A cells^34, 36, 86^, whereas YTHDF1/3 did not show significant translation promotion effect in HEK293T and MCF7 cells^22, 87^. In this study, we confirm translation promotion functions of YTHDF1/3 and provide a mechanism to explain the translation promoting effect of these proteins in different cellular contexts. We show that protein PTM, in particular O-GlcNAcylation on YTHDF1 and YTHDF3, majorly regulates their protein-interaction and granule-formation properties (Figure 7). In our model, high O-GlcNAcylation of YTHDF1/3 (HEK cells) inhibits the translation promotion effect while low O-GlcNAcylation of YTHDF1/3 (HeLa cells) promote the translation of the substrate mRNAs (Figure 7). The O-GlcNAcylation level of YTHDF1/3 is very different in HeLa and HEK293T cells, explaining different results observed when using different cell lines and experimental conditions. The levels of PTMs such as O-GlcNAcylation and protein partners in different cellular contexts will be important to dissect cellular functions of YTHDF1/3 in the future.

In addition, the protein sequences around YTHDF1/3 O-GlcNAcylation is different for YTHDF2; YTHDF2 could not be O-GlcNAcylated to noticeable levels and thus is not likely subjected to this regulation (Figure 7a). With low O-GlcNAcylation in HeLa cells YTHDF1 interacts with ribosome components and promotes translation of its target mRNAs. In HEK293T cells, YTHDF1 is highly O-GlcNAcylated, and this modification prevents its interaction with translation components and thus suppresses its translation-promotion function. Therefore, in this new model, post-translation modifications on YTHDF1/3 critically impact their biological functions.

### YTHDF1 and YTHDF3 differ from YTHDF2 on O-GlcNAcylation

Through systematic mapping protein PTMs using mass spectrometry, we found very similar patterns of PTMs in the YTH domains, where the sequence of all three YTHDF proteins is highly conserved (Figure 1a and S1a), indicating that these YTHDF proteins have a similar function in m^6^A binding. Surprisingly, we discovered that the protein O-GlcNAcylation displays a distinct pattern in the effector domains, with YTHDF1 and YTHDF3 showing high O-GlcNAcylation on the LCD region but not YTHDF2 (Figure 1 and 2). The peptide sequences around these O-GlcNAc modification sites on YTHDF1/3 are different across all three YTHDF proteins, in particular with the corresponding peptide sequences of YTHDF2 (Figure 2a). The abundant O-GlcNAc modification on YTHDF1/3 is located in the LCD region of effector domain, which is known to be involved in protein-protein interaction and condensate formation; the addition of hydrophilic O-GlcNAc modification is thus expected to dramatically alter the biochemical properties of this LCD region. Therefore, YTHDF1 and YTHDF3 but not YTHDF2 are preferred substrates of OGT, despite of their highly consistent overall sequences. Protein O-GlcNAcylation may dramatically tune functions of YTHDF1 and YTHDF3 but not YTHDF2 (Figure 7a).

### O-GlcNAcylation explains cell context-dependent effects of YTHDF1 on translation

Currently, there are two models on functions of YTHDF proteins. One suggests that YTHDF proteins regulate the translation and degradation of m^6^A-modified mRNAs in context-dependent manners. The other argues that YTHDF proteins have a redundant role in mediating mRNA degradation and no translation-promoting effect is detected in YTHDF proteins. Thus, whether YTHDF1/3 promote mRNA translation and why this is cell context dependent remain elusive. Our experimental results support that YTHDF1/3 indeed promote translation of m^6^A modified mRNAs, but found that these promotion effects are regulated by protein O-GlcNAc modification. Biochemical and proteomic analyses demonstrated that the O-GlcNAc modification regulates interaction of YTHDF1 with ribosome components (Figure 3a–d and S4j), with O-GlcNAcylation blocking binding of YTHDF1 with translation machinery (Figure 7b).

O-GlcNAcylation explains the cell-type-dependent effects of YTHDF1 on translation promotion. Our experiments revealed that the higher the level of O-GlcNAc modification, the stronger its inhibitory effect on translation promotion function of YTHDF1/3 (Figure 4a–d, S4a–i). When the stoichiometry of O-GlcNAc modification is high, we not only observe a strong inhibitory effect of translation promotion, but also a decrease in translation efficiency (Figure 4b and S4c). The latter is likely due to the effect of mRNA degradation. Since different cells exhibit distinct levels of O-GlcNAc modification on YTHDF1/3, the existing literatures thus provide conflicting results on this effect in different cell types ^21, 22, 25, 33, 36^. It has been known that YTHDF1 promotes translation in HeLa cells but not HEK293T cells. Our study showed that YTHDF1 is highly O-GlcNAc modified in HEK293T cells, which suppresses its function of translation promotion. The protein level of YTHDF1 is high in HeLa cells but with lower O-GlcNAcylation level, therefore YTHDF1 promotes translation of its target mRNAs. We further showed that dynamic O-GlcNAc fluctuation during cell cycle regulates translation of target transcripts of YTHDF1/3 in a cell-cycle-dependent manner (Figure 5 and S8). The translation efficiency of m^6^A-modified mRNA is much lower in M-phase than in S phase, which is consistent with the much lower overall translational activity of M-phase reported in the literature ^74^ (Figure 7b).

### O-GlcNAcylation also affects properties of YTHDF proteins in granule formation

A remarkable previous study revealed that O-GlcNAc-modified proteins are prominent components of SGs and O-GlcNAcylation of the translational machinery is required for aggregation of untranslated RNP into SGs^88^. Inspired by this study, we then investigated the biological function of YTHDF1/3 O-GlcNAcylation in SGs and revealed that O-GlcNAcylated YTHDF1/3 also participate in SGs and regulate SGs assembly, dynamics, and disassembly. Interestingly, O-GlcNAcylation is known to disfavour granule formation, thereby increasing dynamic nature of YTHDF1/3 in the formation of condensates and trafficking in and out of granules^80, 88, 89^. We showed that O-GlcNAc modification of YTHDF1/3 notably affects the assembly and disassembly of stress granules (Figure 6 and S9–10). This modification could: i) affect dynamics of delivering methylated mRNA by YTHDF1/3 to granules as the O-GlcNAc-modified proteins may not enter the granule; ii) the granule formation as YTHDF1/3 are important components for stress granules. The presence of O-GlcNAc modification would reduce participation of YTHDF1/3 in granule formation; iii) the recovery after stress with increased O-GlcNAc modification promoting the function of YTHDF1 and accelerating the translation restoration (Figure 6l and S9o–p). YTHDF1/3 is thus essential for granules with other RBPs including YTHDF2. The O-GlcNAcylation may notably tune functions of other RNP granules through affecting participation and binding of YTHDF1/3.

In summary, our results revealed a critical regulation pathway for YTHDF1 and YTHDF3 but not YTHDF2. We show that these three YTHDF proteins are different in O-GlcNAc modification. O-GlcNAcylation of YTHDF1 and YTHDF3 regulates their interaction with other proteins and their involvement in granule formation. The level of O-GlcNAcylation controls translation-promotion function of YTHDF1 and YTHDF3. Our study therefore provides the most updated model of functions of YTHDF proteins (Figure 7a). Furthermore, O-GlcNAc has been reported to be a nutrient-sensing PTM that is dynamically regulated by nutritional conditions under various physiological and pathological conditions ^47, 90, 91^. Our study thus directly integrates nutrient-sensing O-GlcNAc modifications with mRNA metabolism and translation through the m^6^A methylation.

## Supplementary Material

Fig 8

Fig 9

Fig 10

Fig 11

Fig 12

Fig 13

Fig 14

Fig 15

Fig 16

Fig 17

SI 1

SI 2

SI 3

SI 4

SI 5

SI 6
