# Supplementary figures and images for: O-GlcNAcylation determines the biological function of YTHDF proteins
Source: Nat Cell Biol. Author manuscript; Available in PMC 2025 May 8. (PMC12060179; doi:10.1038/s41556-023-01258-x)

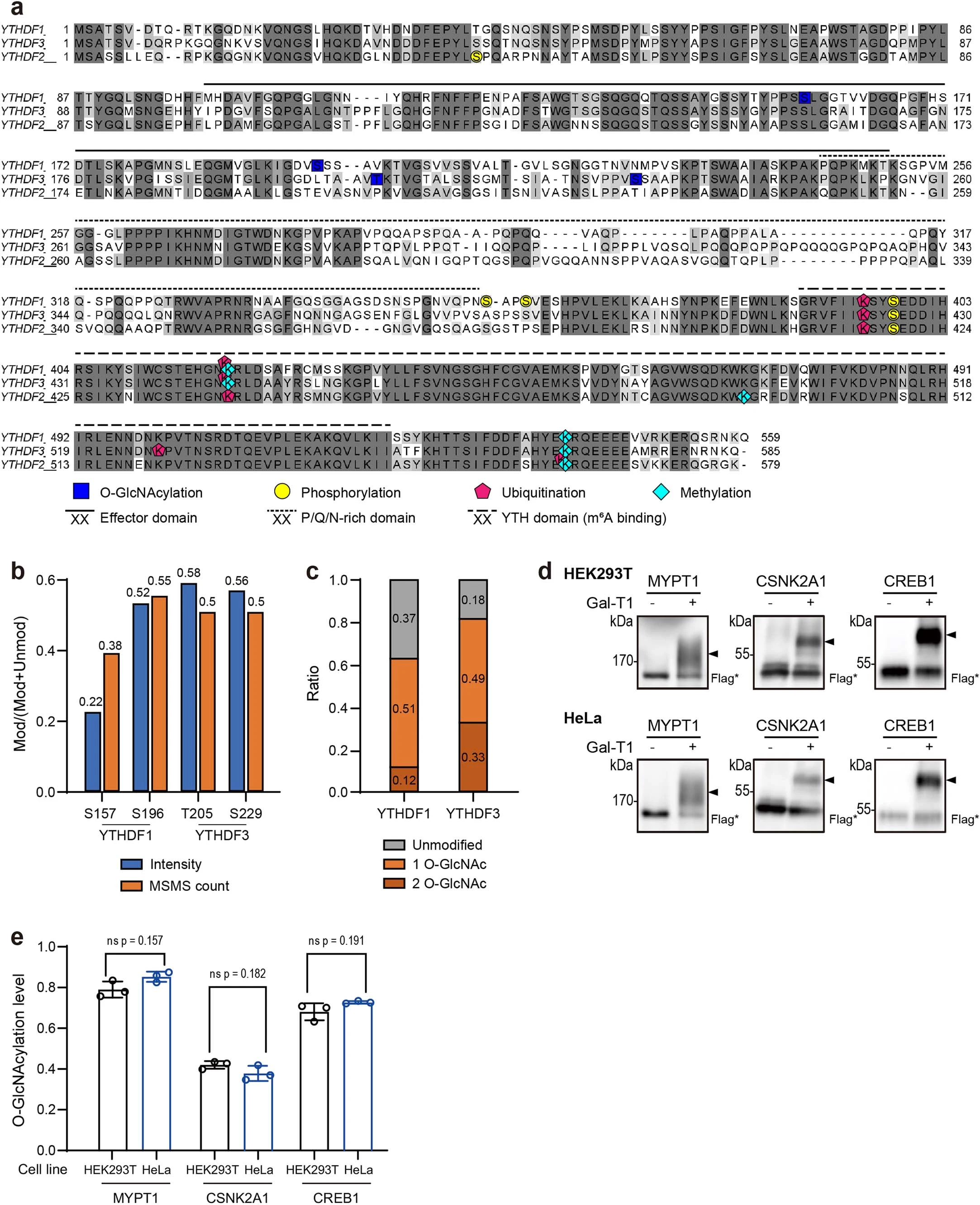

Supplement: Fig 8 [file NIHMS2063183-supplement-Fig_8.webp]

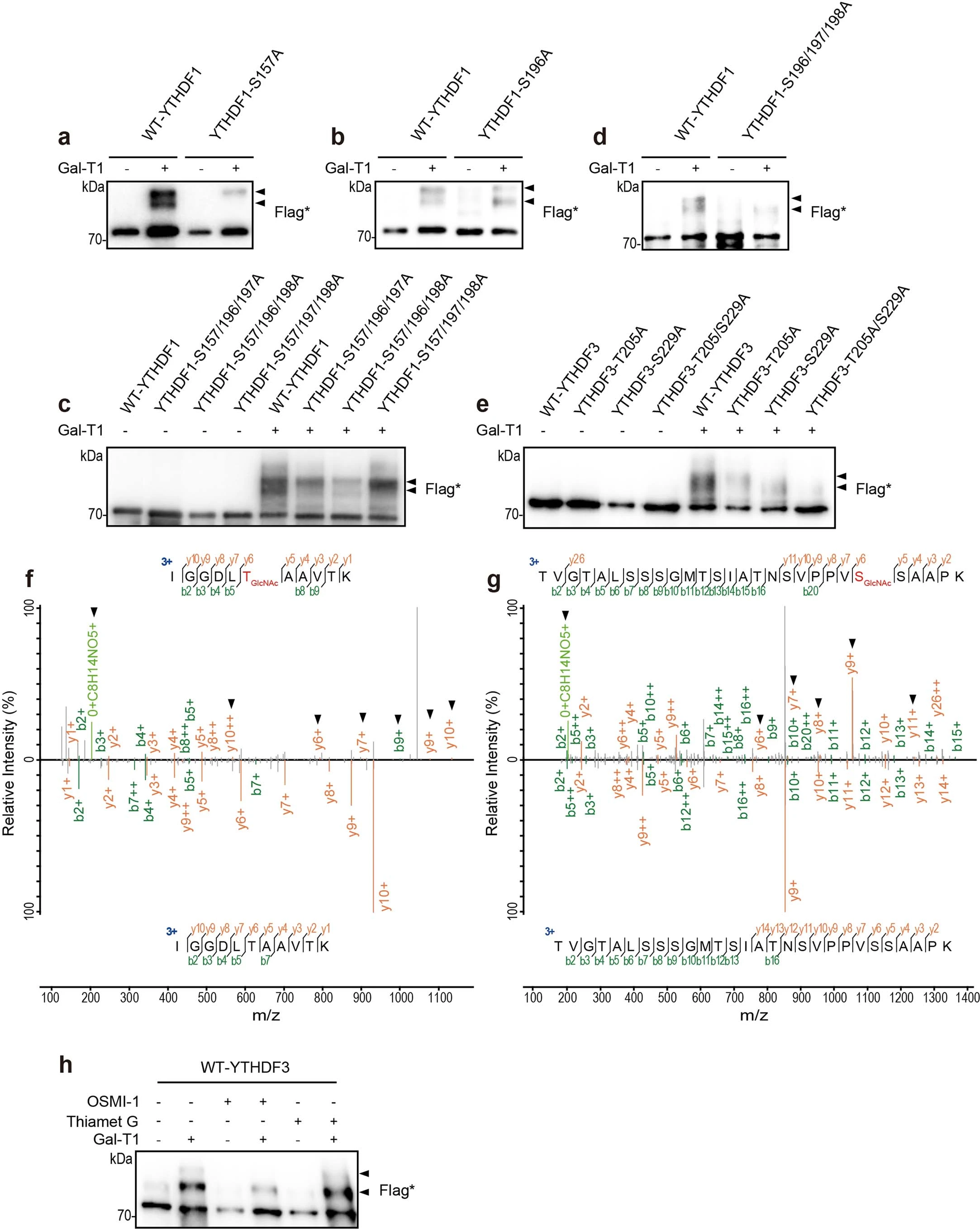

Supplement: Fig 9 [file NIHMS2063183-supplement-Fig_9.webp]

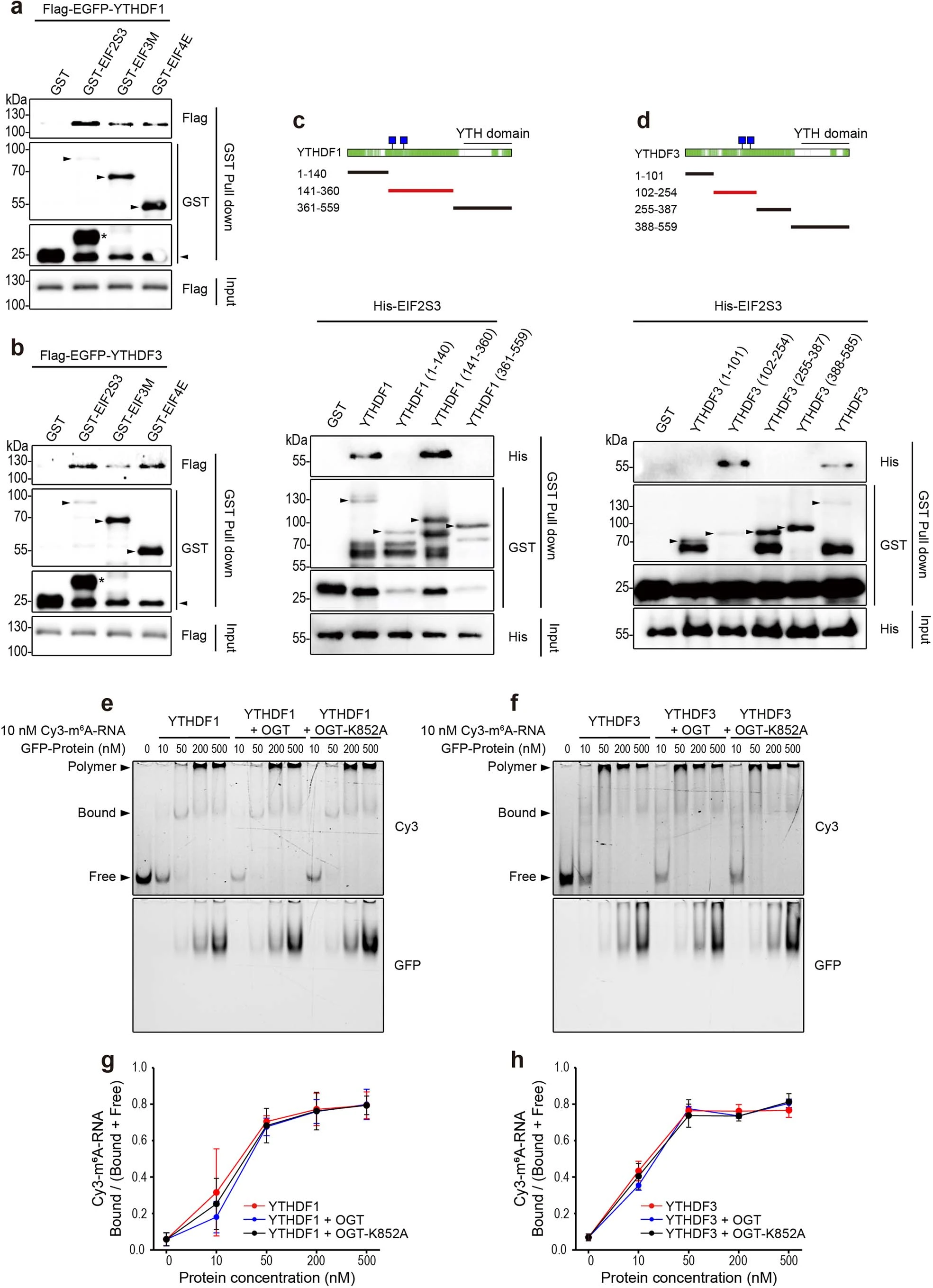

Supplement: Fig 10 [file NIHMS2063183-supplement-Fig_10.webp]

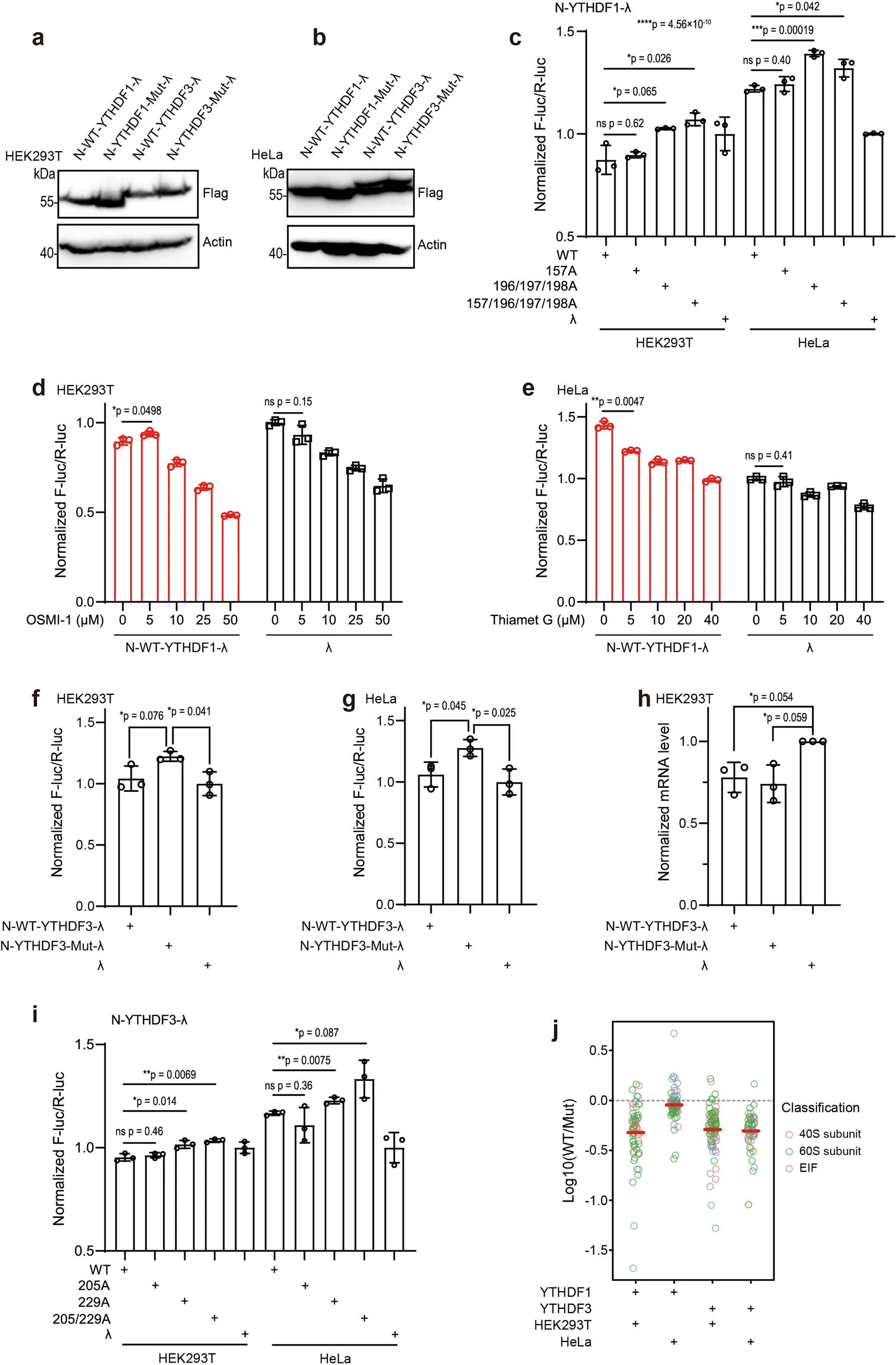

Supplement: Fig 11 [file NIHMS2063183-supplement-Fig_11.webp]

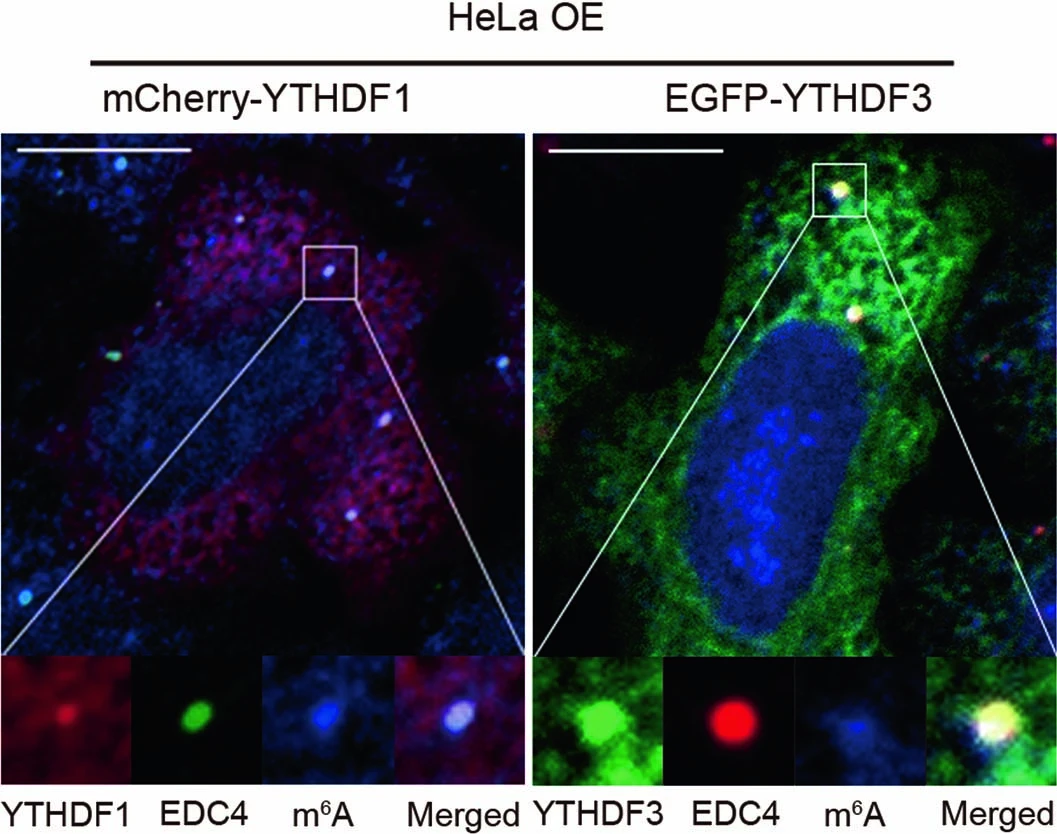

Supplement: Fig 12 [file NIHMS2063183-supplement-Fig_12.webp]

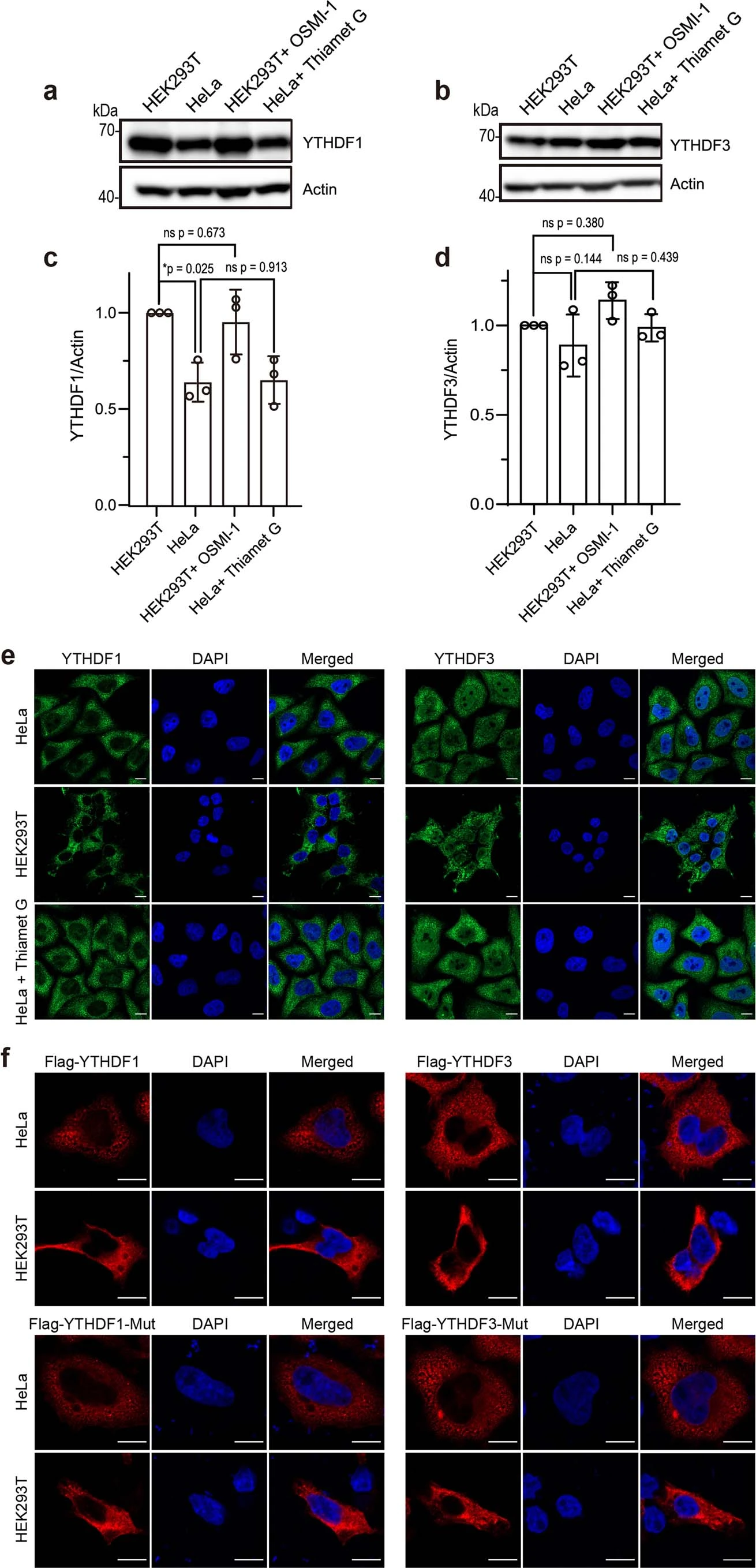

Supplement: Fig 13 [file NIHMS2063183-supplement-Fig_13.webp]

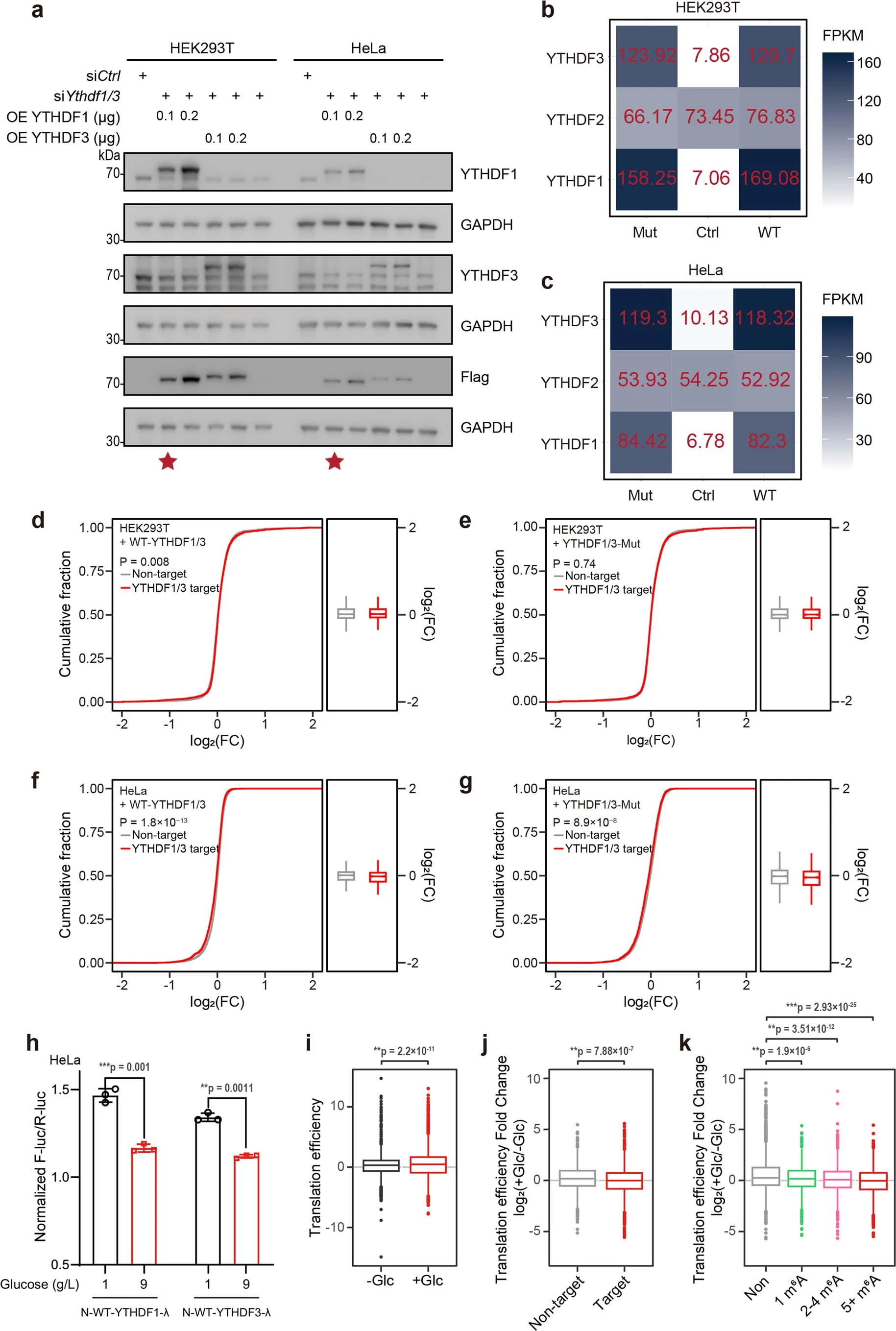

Supplement: Fig 14 [file NIHMS2063183-supplement-Fig_14.webp]

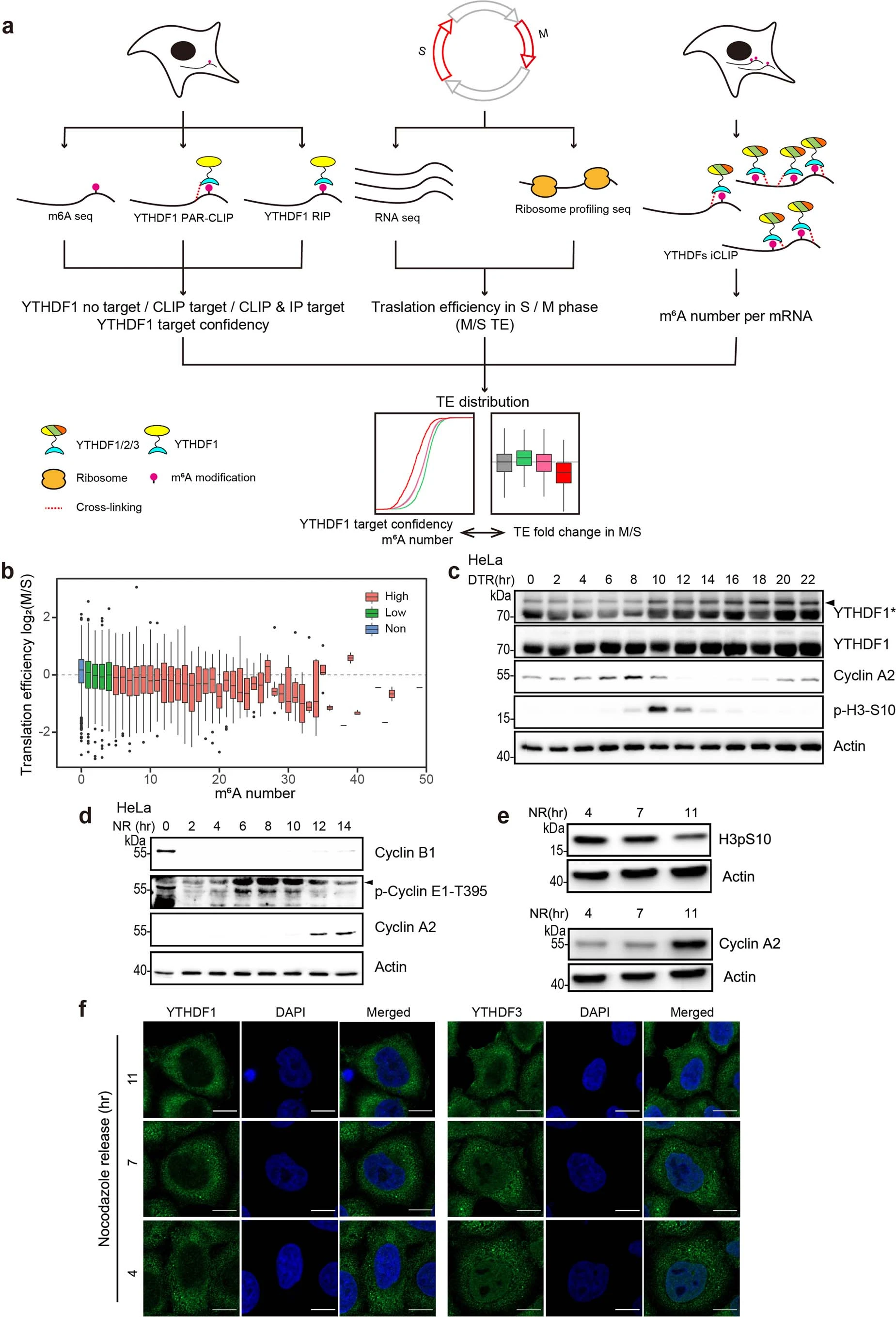

Supplement: Fig 15 [file NIHMS2063183-supplement-Fig_15.webp]

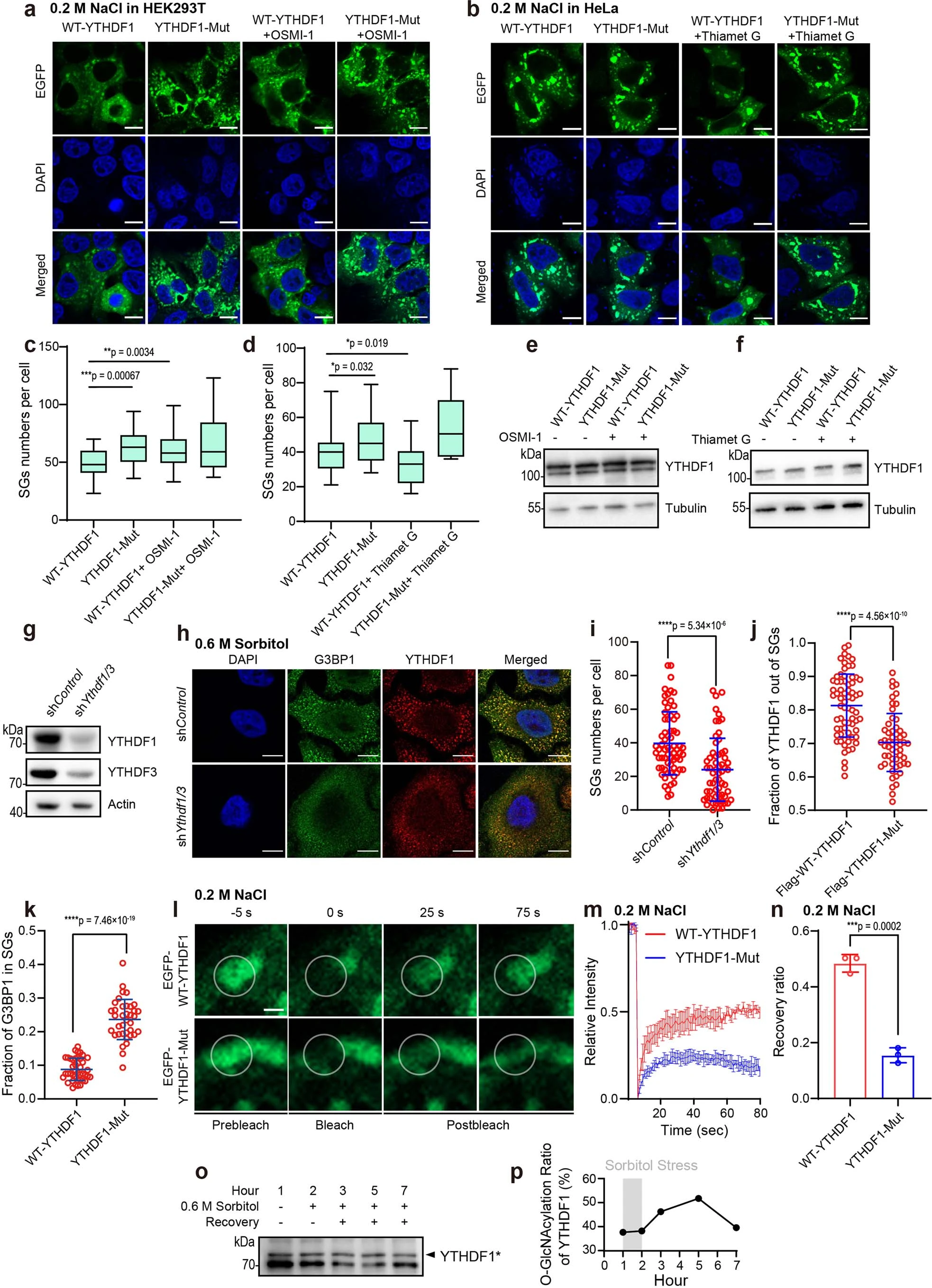

Supplement: Fig 16 [file NIHMS2063183-supplement-Fig_16.webp]

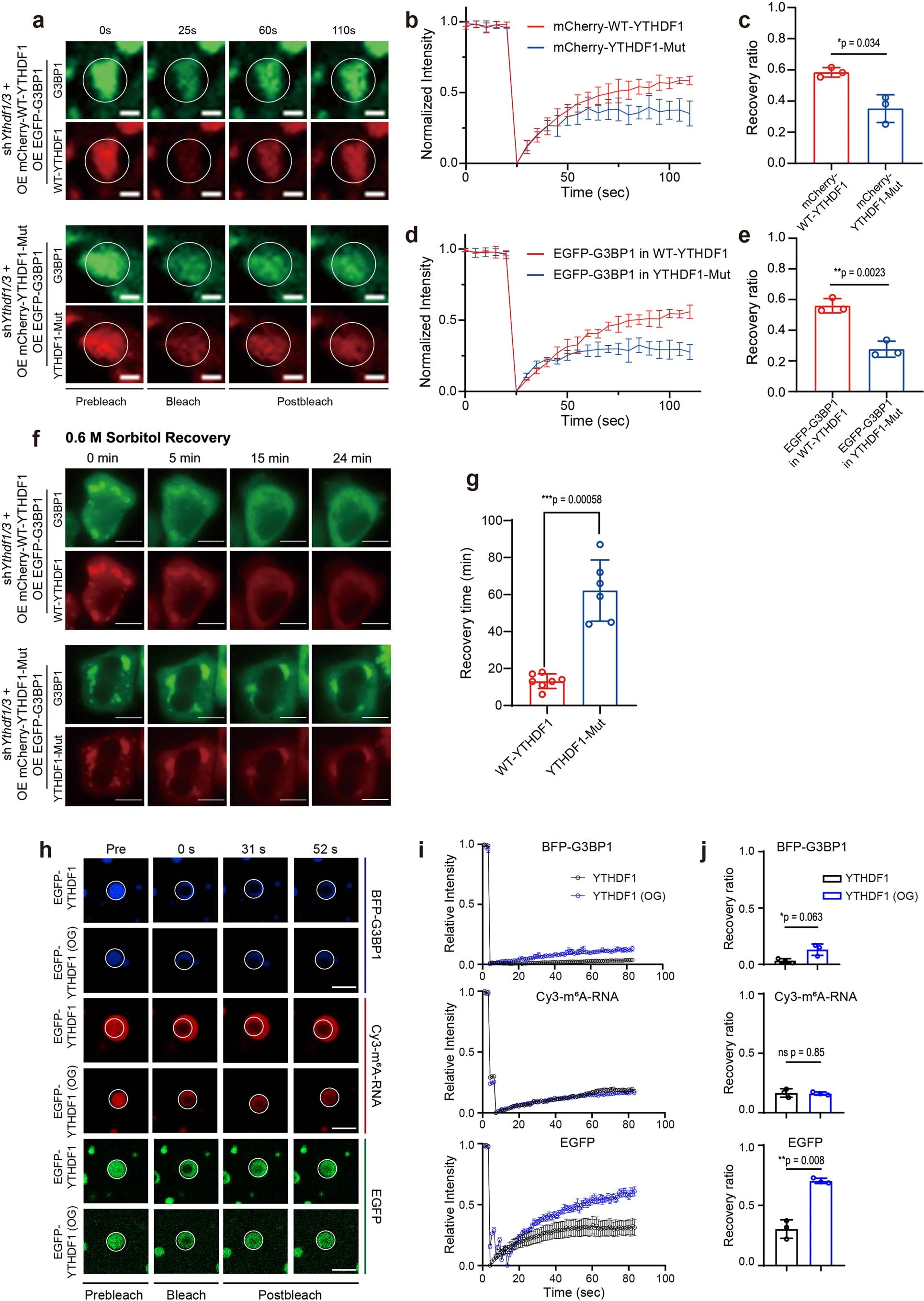

Supplement: Fig 17 [file NIHMS2063183-supplement-Fig_17.webp]
